# eDOL mHealth App and Web Platform for Self-monitoring and Medical Follow-up of Patients With Chronic Pain: Observational Feasibility Study

**DOI:** 10.2196/30052

**Published:** 2022-03-02

**Authors:** Nicolas Kerckhove, Noémie Delage, Sébastien Cambier, Nathalie Cantagrel, Eric Serra, Fabienne Marcaillou, Caroline Maindet, Pascale Picard, Gaelle Martiné, Rodrigue Deleens, Anne-Priscille Trouvin, Lauriane Fourel, Gaelle Espagne-Dubreuilh, Ludovic Douay, Stéphane Foulon, Bénédicte Dufraisse, Christian Gov, Eric Viel, François Jedryka, Sophie Pouplin, Cécile Lestrade, Emmanuel Combe, Serge Perrot, Dominique Perocheau, Valentine De Brisson, Pascale Vergne-Salle, Patrick Mertens, Bruno Pereira, Abdoul Jalil Djiberou Mahamadou, Violaine Antoine, Alice Corteval, Alain Eschalier, Christian Dualé, Nadine Attal, Nicolas Authier

**Affiliations:** 1 Service de Pharmacologie médicale Centre Hospitalier Universitaire de Clermont-Ferrand Clermont-Ferrand France; 2 Centre d'évaluation et de traitement de la douleur Centre Hospitalier Universitaire de Clermont-Ferrand Clermont-Ferrand France; 3 Centre d'évaluation et de traitement de la douleur Centre Hospitalier Universitaire de Toulouse Toulouse France; 4 Centre d'évaluation et de traitement de la douleur Centre Hospitalier Universitaire d'Amiens Amiens France; 5 Centre d'évaluation et de traitement de la douleur Centre Hospitalier Universitaire de Grenoble Grenoble France; 6 Centre d'évaluation et de traitement de la douleur Centre Hospitalier Universitaire de Limoges Limoges France; 7 Centre d'évaluation et de traitement de la douleur Centre Hospitalier Universitaire de Rouen Rouen France; 8 Centre d'évaluation et de traitement de la douleur Assistance Publique–Hôpitaux de Paris Cochin Paris France; 9 Centre d'évaluation et de traitement de la douleur Centre Hospitalier Régional de Bayeux Bayeux France; 10 Centre d'évaluation et de traitement de la douleur Hospices Civils de Lyon Pierre Wertheimer Lyon France; 11 Centre d'évaluation et de traitement de la douleur Centre Hospitalier Universitaire de Nîmes Nîmes France; 12 Laboratoire d'Informatique, de Modélisation et d'Optimisation des Systèmes Université Clermont Auvergne Aubière France; 13 Institut Analgesia Clermont-Ferrand France; 14 Institut National de la Santé et de la Recherche Médicale, Unité 987 – Centre d'évaluation et de traitement de la douleur Assistance Publique–Hôpitaux de Paris Ambroise Paré Paris France

**Keywords:** mHealth, chronic pain, feasibility study, eHealth, self-monitoring

## Abstract

**Background:**

Chronic pain affects approximately 30% of the general population, severely degrades quality of life (especially in older adults) and professional life (inability or reduction in the ability to work and loss of employment), and leads to billions in additional health care costs. Moreover, available painkillers are old, with limited efficacy and can cause significant adverse effects. Thus, there is a need for innovation in the management of chronic pain. Better characterization of patients could help to identify the predictors of successful treatments, and thus, guide physicians in the initial choice of treatment and in the follow-up of their patients. Nevertheless, current assessments of patients with chronic pain provide only fragmentary data on painful daily experiences. Real-life monitoring of subjective and objective markers of chronic pain using mobile health (mHealth) programs can address this issue.

**Objective:**

We hypothesized that regular patient self-monitoring using an mHealth app would lead physicians to obtain deeper understanding and new insight into patients with chronic pain and that, for patients, regular self-monitoring using an mHealth app would play a positive therapeutic role and improve adherence to treatment. We aimed to evaluate the feasibility and acceptability of a new mHealth app called eDOL.

**Methods:**

We conducted an observational study to assess the feasibility and acceptability of the eDOL tool. Patients completed several questionnaires using the tool over a period of 2 weeks and repeated assessments weekly over a period of 3 months. Physicians saw their patients at a follow-up visit that took place at least 3 months after the inclusion visit. A composite criterion of the acceptability and feasibility of the eDOL tool was calculated after the completion of study using satisfaction surveys from both patients and physicians.

**Results:**

Data from 105 patients (of 133 who were included) were analyzed. The rate of adherence was 61.9% (65/105) after 3 months. The median acceptability score was 7 (out of 10) for both patients and physicians. There was a high rate of completion of the baseline questionnaires and assessments (mean 89.3%), and a low rate of completion of the follow-up questionnaires and assessments (63.8% (67/105) and 61.9% (65/105) respectively). We were also able to characterize subgroups of patients and determine a profile of those who adhered to eDOL. We obtained 4 clusters that differ from each other in their biopsychosocial characteristics. Cluster 4 corresponds to patients with more disabling chronic pain (daily impact and comorbidities) and vice versa for cluster 1.

**Conclusions:**

This work demonstrates that eDOL is highly feasible and acceptable for both patients with chronic pain and their physicians. It also shows that such a tool can integrate many parameters to ensure the detailed characterization of patients for future research works and pain management.

**Trial Registration:**

ClinicalTrial.gov NCT03931694; http://clinicaltrials.gov/ct2/show/NCT03931694

## Introduction

Chronic pain affects approximately 30% of the general population [1-6] and was 1 of the top 5 leading causes of years lived with disability in 2016 [7], especially among older people [8]. Societal and economic issues are also crucial, as 60% of people with chronic pain are less able or unable to work, and 20% report having lost their job as a result [9]. The overall cost of chronic pain is estimated to be approximately €441 billion in Europe (equivalent to approximately US $496 billion) and $560 to $635 billion in the United States [10-12]. At the same time, the market for analgesic drugs represented approximately $68 billion in 2016, and an increase from 2% to 5% was forecast for 2021, with a further 5% increase by 2025 [13,14]. Unfortunately, available analgesics are old, their effectiveness is limited, with undesirable effects, and little progress has been made in recent years [15]. Thus, innovation is limited despite prolific basic research [16].

Various reasons are given for this, including the relevance of animal research [17]. In particular, because of the low success rate of validation of preclinical concepts during the transition to the clinic. Developments in this area could help progress, but such progress could also come from better patient characterization that would help to identify the predictors of successful treatments through research programs and enable physicians to carry out better decision-making regarding the initial choice of treatment and its follow-up. Subgroups of patients and criteria for response to particular treatments, for example, in patients with neuropathic pain [18], have been identified; however, such characterization should not be limited to biomedical assessment but should also include biopsychosocial assessment. Moreover, current assessments of patients with chronic pain provide only fragmentary data on daily experiences because of recall bias. Thus, it is essential to modify the temporality in which patients’ sensations are assessed, with real-life monitoring of subjective and objective markers of chronic pain. This strategy is currently being developed by several research teams evaluating smartphone apps or web platforms for use in managing the treatment of patients with chronic pain [19-24].

We hypothesized that regular self-monitoring by patients using a digital app would generate in-depth knowledge and new insights for physicians, and would allow patients to be active in their own care and benefit from web-based counseling. Regular self-monitoring would not only contribute to better patient characterization and help in choosing the most appropriate treatment but may also improve adherence to treatment. Moreover, recent studies [19,20,24-31] have highlighted the urgent need to develop eHealth self-monitoring programs for chronic pain and their therapeutic value—web-based pain management programs (The Pain Course) based on principles of cognitive behavior therapy were found to be beneficial for patients by reducing pain symptoms and associated comorbidities [20,32-34], and there is therapeutic interest in mobile health (mHealth) technologies for managing the medical treatments of patients suffering from chronic pain [27]. In this pilot study, we aimed to evaluate the feasibility and acceptability of a new mHealth app and web platform, called eDOL, for patients and physicians.

## Methods

### Ethics

The study was approved by the *Comité de Protection des Personnes Ile de France V* (2018-A01790-5546) and is registered (NCT03931694). The study was conducted in accordance with French laws and regulations on research on human beings and data protection and with the Declaration of Helsinki [35].

### Confidentiality and Data Entry and Processing

Data were collected and managed using the eDOL app, developed by Bepatient and hosted by Avenir Télématique. In accordance with the provisions relating to the confidentiality of information concerning, in particular, the people who took part in the research and the results obtained [36], individuals with direct access have taken all the necessary precautions to ensure the confidentiality of the information relating to the participants. These persons and the investigators themselves are subject to professional secrecy [37]. All data collected and transmitted to the sponsor (University Hospital of Clermont-Ferrand) were anonymized, and each patient had a single coded number. The head of research ensured that each patient was informed of which data were collected and that they did not object to their use or disclosure.

Answers to questionnaires and medical data were transmitted in spreadsheet format (Excel 2013, Microsoft Inc). All anonymized data were accessible to the biostatisticians (BP, SC, and AJD), the coordinator (ND), and the project manager (NK). Only the investigators could access their patients' personal data to identify them. A dashboard linking patients’ identities and study IDs was available only on the investigators' professional interface on the eDOL web platform. The final database, used for statistical analyses, included only study IDs to preserve anonymity.

### Study Design and Population

To evaluate the feasibility and acceptability of the eDOL app for the characterization, real-life monitoring of patients with chronic pain from 12 pain clinics in France took place between February 8, 2019 and January 8, 2020. The study was offered to all physicians in the investigating centers.

Participation in the study was offered to patients with chronic pain who did not have cancer, who were owners and regular users of a smartphone, and who were followed up in a pain clinic. All adult (≥18 years old) patients able to read and understand French and provide consent to participate in the study were included (with a yes-or-no choice on the eDOL app). Participants were free to withdraw their consent at any time by informing the sponsor. Each patient had access to the information document (paper or electronic) detailing the purpose, content, and conduct of the study. If they agreed to participate, they were asked to download the eDOL app and complete the questionnaires using the eDOL app. The URL to access this app was sent by email from physicians to their patients. After downloading the app and creating their profile, patients could accept the general terms and conditions of use and confirm that they agree to the use of their medical data in this study.

Each patient had 1 initial study visit, during which, the physician introduced the study to the patient, checked their eligibility, explained the eDOL tool, and gave the patient a brief training document on how to use the eDOL smartphone app. Participants completed several questionnaires and assessments using the eDOL app over a period of 2 weeks (initial patient characterization) and then repeatedly over a period of 3 months and up to 6 months for patients who wished to continue using the app (weekly, quarterly, and half-yearly depending on the questionnaire). Physicians saw their patient at a follow-up visit that took place at least 3 months after the inclusion visit, with the possibility of continuing the follow-up for up to 6 months. The study was considered complete for patients who completed their questionnaires and assessments for at least 3 months and made a follow-up visit 3 to 6 months after the inclusion visit.

### eDOL App

All data were collected using the eDOL digital health tool, which includes a smartphone app for patients that allows self-questionnaires and assessments to be completed for semiological monitoring (pain, anxiety, sleep quality), and a web interface for physicians, to allow them to graphically visualize the summary of data provided by their patients for clinical and therapeutic monitoring.

Patients completed questionnaires and weekly assessments (Multimedia Appendix 1). The questionnaires were divided into general questionnaires that were systematically filled in once only (sociodemographic, lifestyle and professional data, Pain Beliefs and Perceptions Inventory [38]; Evaluation of level of precariousness [39]; Injustice Experience Questionnaire [40]; Maslach Burn-out Inventory [41]; Toronto Alexithymia Scale [42]; Life Orientation Test-Revised [43]; Belief in a just world [44]; Job Content Questionnaire [45]; Big Five Inventory [46]) and questionnaires, assessing symptoms, comorbidities, and psychological and physiological states related to chronic pain, that were completed quarterly (Brief Pain Inventory [47]; Medical Outcomes Study Sleep Scale [48]) and, according to duration of follow-up, half-yearly (Tampa Scale of Kinesiophobia [49]; Pain Catastrophizing Scale [50]; Fear-avoidance beliefs [51]; EQ-5D-3L [52]; Hospital Anxiety Depression Scale [53]; Satisfaction With Life Scale [54]; Subjective Cognitive Complaints [55]). Some questionnaires were specific to a type of chronic pain (Neuropathic Pain Scale Inventory [56]; Western Ontario and McMaster Universities [57]; Rheumatoid Arthritis Impact of Disease [58]; Roland Morris Disability Questionnaire [59]; Irritable Bowel Severity Scoring System [60]; Fibromyalgia Impact Questionnaire [61]; Headache Impact Test [62]). Follow-up of patients (daily monitoring of various objective and subjective parameters related to the pathology), using assessments, was also integrated in the app, which allowed us to monitor the evolution of patients' pain and its repercussions. Assessments were in the form of an 11-point numeric rating scale (from 0 to 10), assessing the intensity of pain (average, minimum, or maximum intensity), anxiety, fatigue, and the quality of sleep, morale, body comfort were assessed weekly for 3 to 6 months.

For physicians, the eDOL internet platform included a simple and ergonomic dashboard which allowed the physician to find all of their patients included in the study, with the following tabs: (1) Management, in which all of the medical records completed by the physician could be found (history, pain diagnosis, initial characterization, next appointment, consultation sheets and treatment sheets); (2) Health Measures, which showed a graphic display of the real-life follow-up of all the weekly assessments; and (3) Questionnaires, which showed all the questionnaires completed by the patients (display of questionnaire scores and answers to all the questions). The eDOL platform enabled physicians to complete medical elements during consultation visits with various medical form (diagnosis, current treatments, examination results). The physicians could also activate new questionnaires to be filled in by their patients, either to complete the characterization (eg, specific questionnaires for pain diagnosis) or to evaluate other criteria (eg, evaluation of the Patients’ Global Impression of Change after the introduction of a new treatment [63]). Diagnostic questionnaires (Posttraumatic stress disorder Checklist [64]; Neuropathic pain 4 [65]; Fibromyalgia Rapid Screening Tool [66]), reminders of the criteria for diagnoses (ROME IV for irritable bowel syndrome; Widespread pain index and Symptom severity scale of American college of rheumatology for fibromyalgia; Neuropathic Pain IASP Special Interest Groups for neuropathic pain), and screening tools for opioid misuse (Prescription Opioid Misuse Index [67] and Opioid Risk Tool [68]) were also at their disposal (Table 1).

**Table 1:** eDOL features.

Feature	Included in	Assessment point or interval	Details
Inclusion form	Investigator web platform	Initial visit	Last name, first name, email, ID number
		Initial visit	History (clinical, psychiatric, drug), clinical examination, medico-economic aspect (type of medical consultations), diagnosis of pain according to International Classification of Disease, 11th revision
Personal information	Smartphone app	Initial visit	Sociodemographic (work, alcohol use, tobacco use)
		Initial visit	Pain characterization: frequency, duration, aggravating and alleviating factors
Treatment forms	Investigator web platform	Updated at each consultation	Analgesics (name, dates, dosage, side effects); list of nonmedicinal techniques and other treatments (free text)
Assessments	Smartphone app	Repeated weekly	11-point numeric rating scale (0-10): sleep, morale, fatigue and energy, body comfort, anxiety, pain
Self-questionnaires	Smartphone app	During the first 2 weeks	5 sessions of questionnaires
		Not repeated	Fear-avoidance beliefs^a^, Injustice Experience Questionnaire, Maslach Burn-out Inventory^a^, Pain Beliefs and Perceptions Inventory, Evaluation of level of precariousness, Job Content Questionnaire^a^, Life Orientation Test-Revised, Belief in a just world, Posttraumatic Stress Disorder Checklist^b^, Toronto Alexithymia Scale Big Five Inventory
		Every 3 months	Fibromyalgia Impact Questionnaire^c^, Headache Impact Test^c^, irritable bowel severity scoring system^c^, Prescription Opioid Misuse Index^b^, Patients’ Global Impression of Change^b^, Neuropathic Pain Scale Inventory^b^, Rheumatoid Arthritis Impact of Disease^b^, Brief Pain Inventory, Medical Outcomes Study Sleep Scale
		Every 6 months	Tampa Scale of Kinesiophobia, Roland Morris Disability Questionnaire^c^, Western Ontario and McMaster Universities^c^, Pain Catastrophizing Scale, EuroQol 5 dimensions 3 levels, Hospital Anxiety Depression Scale, Satisfaction With Life Scale, Subjective Cognitive Complaints
Hetero-questionnaires	Investigator web platform	N/A^d^	Diagnostic validation: Neuropathic pain 4 + NEUPSIG (neuropathy), Widespread pain index and Symptom severity scale and Fibromyalgia Rapid Screening Tool (fibromyalgia), ROME IV (irritable bowel syndrome)
		Updated at each consultation	Others: Opioid Risk Tool
Consultation form	Investigator web platform	Updated at each consultation	clinical examination, medico-eco aspect, observance, benefit-risk ratio of treatments

^a^Work-related questionnaires.

^b^Optional questionnaires.

^c^Disease-specific questionnaires

^d^N/A: not applicable.

### Study Outcomes

The primary study endpoint reflected the acceptability of the eDOL app and the feasibility of its use and was assessed with a satisfaction survey (based on the Patient Satisfaction Questionnaire Short Form [69] and the Client Satisfaction Questionnaire [70,71]) for patients (10 questions) and for participating physicians (12 questions) at the end of the study. The satisfaction survey (in French language) was sent to each patient 6 months after their inclusion visit and was sent to the physicians after the last patient follow-up, via the eDOL tool. Response options for each question ranged from 0 (strongly disagree with the statement) to 10 (strongly agree with the statement). A mean score of at least 7 out of 10 was considered to reflect satisfactory acceptability and feasibility of the eDOL tool. The questionnaire completion rate and center participation (inclusion rate) were also calculated.

Secondary analyses to characterize participating patients, pain disorders, and related comorbidities, as well as clustering analysis of the participants to determine the profile determination of patients who adhered to the use of the app were undertaken to gain insight into the capabilities and added value of the tool for the characterization and the follow-up of patients with chronic pain.

### Statistics

#### Sample Size

A minimum of 100 patients were to be included and analyzed. Such a large number of patients is quite satisfactory in terms of descriptive analyses to study the feasibility of a multimodal eHealth tool. This number of patients is in line with that specified by Sundararaman et al [27] and those used in other recent studies [19,23,24,72] evaluating smartphone apps in patients with chronic pain. This number of patients (n=100) allowed us to analyze in an exploratory way: (1) the characterization of patients with chronic pain followed-up in pain clinics, (2) the description and understanding of their pain, (3) the multiple dimensions and the numerous neuropsychiatric repercussions of chronic pain, and (4) the clustering of the participants and the determination of adhering patients’ profiles.

#### Statistical Analysis

We performed statistical analyses to determine if patients and physicians were satisfied with the tool and adhered to its use, and to identify interesting pain profiles of patients, and which profiles are most adherent (and for how long).

Patients were described according to epidemiological characteristics, clinical characteristics, and treatment characteristics. The key indicators for acceptability (patient and physician) were questionnaire completion and completion of follow-up medical forms. We determined the association between adherence and all baseline variables. A patient was defined as adherent if 100% of baseline questionnaires and 75% of assessments after 3 months follow-up were completed.

Continuous variables and scale variables (treated as ordinal data) were presented as mean and standard deviation (for normal distributions), or median and quartiles (for asymmetric distributions). The normality assumption was assessed with graphical criterion and the Shapiro-Wilk test. Categorical variables were expressed in number and percentage.

We performed clustering analysis. Patients were clustered according the symptoms and comorbidity information y. This clustering analysis included data imputation, principal component analysis of baseline data, and ascending hierarchical classification gathering 90% of total inertia. From these components, the hierarchical classification [73] in Euclidean coordinates was used.

Comparisons (baseline vs 3-month follow-up, by patient adherence, and by cluster) were performed using the chi-square test or Fisher exact test when assumptions to apply chi-square were not met (minimal level of expected number of cases under independence assumption), for categorical variables, and using analysis of variance (or Kruskal-Wallis tests when the assumptions to apply analysis of variance were not met). When the omnibus *P* value was statistically significant (*P*<.05), posthoc tests (independent *t* test or Mann-Whitney) were applied to compare subgroups with each other. The results were expressed using effect sizes (Cramer *V* for categorical data and eta square for quantitative data) with 95% confidence intervals. Pearson (preferred between all distributions acknowledged as Gaussian) or Spearman correlation coefficients (otherwise) were calculated depending on the nature of the distribution.

We used Stata (version 15, StataCorp LLC) and R (version 4.0.3) software. All statistical tests were 2-sided with type I error set at 5%.

## Results

### Study Population

Of 133 patients from 12 French pain clinics, 28 patients (28/133, 21.0%) did not install the eDOL app; data from 105 patients were analyzed. The first patient was enrolled on February 6, 2019, and the last patient was enrolled on October 31, 2019.

At baseline, participating patients were mostly middle-aged women, in a couple, nonsmoking, and professionals. Among these patients, 35.3% (30/85) were in work stoppage due to their chronic pain. A more detailed characterization of the patients, with the help of several validated questionnaires, mainly showed that a significant number were considered precarious (43.0%; 40/93), with kinesiophobia (72.0%; 67/93), alexithymia (51/100, 51%), degraded life satisfaction (51/92, 55.4%), catastrophism (47/100, 47.0%) and a possible cognitive disorder (77/93, 82.8%). More than 65% (63/94, 67.0%) of patients had impaired sleep, and 37.2% (35/94) and 27.7% (26/94) had proven anxiety or depressive disorders respectively.

Regarding the characterization of pain disorders and their treatments, most patients (76/83, 91.6%) had moderate to severe pain intensity, of which 20.5% (17/83) had a high chronic pain interference score (called “high impact chronic pain” [74]). Most patients (50/80, 62.5%) suffered from nociplastic pain, with a duration longer than 5 years for more than 50% (55/105, 52.4%) of patients. The majority of patients (56/105, 53.3%) described their chronic pain as permanent (with painful paroxysms every day and lasting >2 hours) and inducing frequent nocturnal awakenings (45/105, 42.8%). Finally, analgesic treatments used by the patients were mainly antidepressants followed by weak opioids (with or without paracetamol), and antiepileptics to a lesser extent. In parallel, 89.2% (66/74) of patients used nonmedicinal analgesic treatments.

There was no difference in any of these characteristics between baseline and the 3-month follow-up (Multimedia Appendix 2).

### Primary Objective: Feasibility and Acceptability

**Figure 1:**
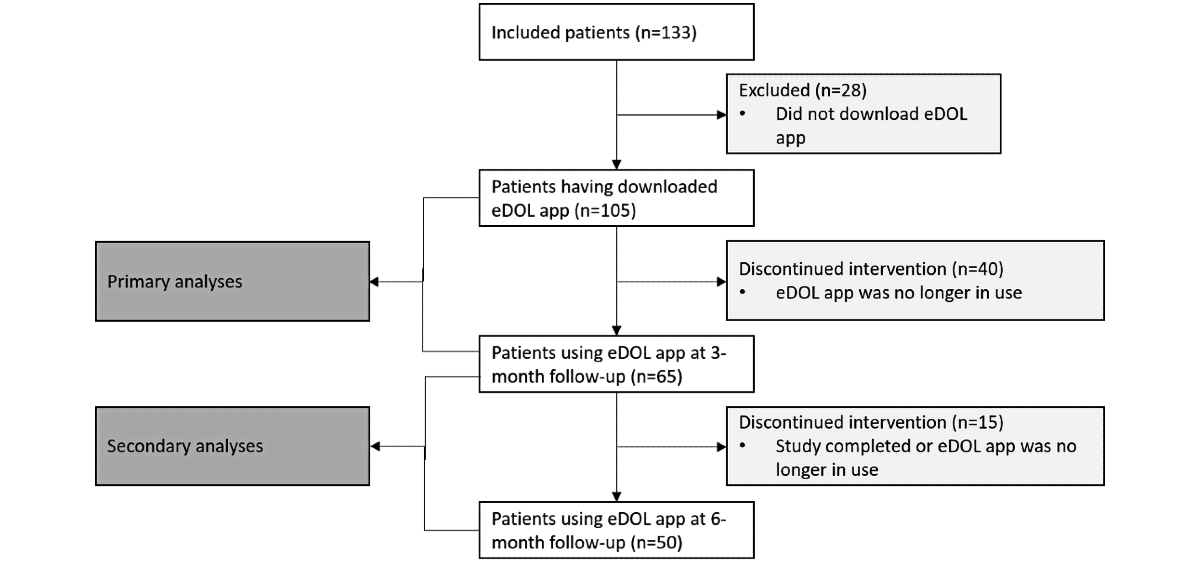
Study flowchart.

Among 105 patients, 65 (61.9%) adhered to the use of the eDOL tool and 50 patients continued using the eDOL tool up to 6-month follow-up (Figure 1).

**Figure 2:**
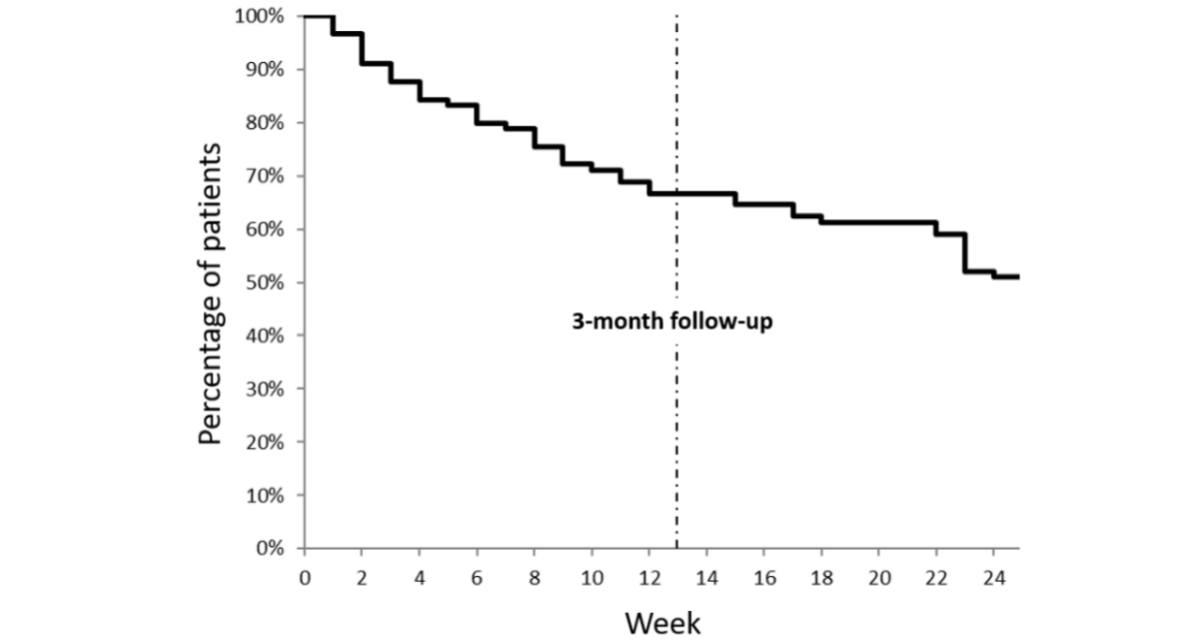
Completion rate over time.

In detail, the overall rate of patient who completed the baseline questionnaires was 89.3% (range 79.0%-95.2%). The quarterly questionnaires, Brief Pain Inventory and Medical Outcomes Study Sleep Scale, were repeatedly filled at 3-month follow-up by 63.8% (67/105) of patients. For the half-yearly questionnaires (Tampa Scale of Kinesiophobia; Pain Catastrophizing Scale; EQ-5D-3L; Hospital Anxiety Depression Scale; Satisfaction With Life Scale and Subjective Cognitive Complaints), 58.7% (range 53.8%-63.1%) of patients completed the questionnaires. The filling rate of the weekly assessments for the real-life monitoring of the different parameters (pain, moral, anxiety, fatigue, sleep and body comfort) was 88.6% (93/105) of patients at the end of the first week and 61.9% (65/105) at 3-month follow-up (Table 2; Figure 2; Multimedia Appendix 3). Due to the small number of patients, we did not show the results concerning the specific questionnaires, filled by only a few patients according to their professional situation (questionnaires on work) and their type of pain (disease-specific questionnaires). The rate of patients whose various medical follow-up forms were completed by the investigators (inclusion, treatment and consultation) was 70.7% (range 62.9-76.2%) (Table 2).

**Table 2:** Questionnaire completion.

Assessment			Baseline (n=105), n (%)		3-month follow-up (n=105), n (%)		6-month follow-up (n=65), n (%)
**Physician baseline and follow-up forms**							
	Inclusion form (baseline)	77 (73.3)		N/A^a^		N/A	
	Diagnosis form (baseline)	80 (76.2)		N/A		N/A	
	Treatment form (baseline and follow-up)	74 (70.5)		N/A		N/A	
	Consultation form (follow-up)	66 (62.9)		N/A		N/A	
**Self-administered questionnaires and assessments**							
	Weekly assessments	93 (88.6)		65 (61.9)		50 (76.9)	
	Toronto Alexithymia Scale	100 (95.2)		N/A		N/A	
	Injustice Experience Questionnaire	100 (95.2)		N/A		N/A	
	Pain Beliefs and Perceptions Inventory	92 (87.6)		N/A		N/A	
	Life Orientation Test-Revised	94 (89.5)		N/A		N/A	
	Belief in a just world	94 (89.5)		N/A		N/A	
	Evaluation of level of precariousness	93 (88.6)		N/A		N/A	
	Big Five Inventory	92 (87.6)		N/A		N/A	
	MOS-Sleep Scale	94 (89.5)		67 (63.8)		39 (60.0)	
	Brief Pain Inventory	93 (88.6)		67 (63.8)		38 (58.5)	
	Pain Catastrophizing Scale	100 (95.2)		N/A		40 (61.5)	
	Satisfaction With Life Scale	92 (87.6)		N/A		35 (53.8)	
	Subjective Cognitive Complaints	93 (88.6)		N/A		35 (53.8)	
	EQ-5D-3L	83 (79.0)		N/A		36 (55.4)	
	Hospital Anxiety Depression Scale	94 (89.5)		N/A		41 (63.1)	
	Tampa Scale of Kinesiophobia	93 (88.6)		N/A		41 (63.1)	

^a^N/A: not applicable.

Among the 12 pain clinics participating in the study, 10 (83.3%) included patients, and 2 withdrew from participation before the start of the study. The median inclusion number per center was 8 (IQR 5.0, 14.0) patients. The inclusion objective (at least 100 analyzable patients) was achieved in less than a year as requested from the investigating centers.

The satisfaction questionnaire was filled in by 65.7% (69/105) of patients at the end of the study. The median acceptability score was 7.0 (IQR 6.1, 7.6), with only 9.5% (10/105) of the patients providing a rating less than 5.0 out of 10. Moreover, 88.6% (93/105) of the patients who responded wanted to participate in the further development of the eDOL app. The items with the lowest scores corresponded to the patients’ perception of the physicians’ use of eDOL in their follow-up (mean 5.7, SD 3.1), patients’ perception of the potential positive impact of eDOL on their pain management (mean 5.8, SD 2.7), and quality of life (mean 5.6, SD 2.4).

A total of 21 physicians participated in the study and included at least one patient, and 15 (71.4%) answered the satisfaction questionnaire. The physicians were mostly women (14/21, 66.7%), approximately 50.1 years old (range 33-61), and were from various specialties (2 neurologists, 2 psychiatrists, 3 anesthesiologists, 3 rheumatologists, and 5 general practitioners). The median acceptability score was 7.2 (IQR 6.8, 8.3), with only 6.7% (1/15) of physicians rating less than 5.0 out of 10. The items with the lowest scores corresponded to the compatibility of eDOL with the electronic medical file systems (mean 5.0, SD 2.3) and the possibility of eventually replacing the electronic medical files with the eDOL tool (mean 4.4, SD 1.9) (Table 3).

**Table 3:** Physician and patient acceptability of eDOL.

Acceptability questionnaire			Score (out of 10), mean (SD)
**Physician**			
	The training and support provided was sufficient to use eDOL correctly	7.3 (1.4)	
	After the first training session, it is easy to use eDOL on a daily basis	6.9 (2.3)	
	The technical support (email and phone) was available to assist me if needed	8.3 (1.2)	
	eDOL offers questionnaires and assessments adapted to the multidimensional characterization of my patients	8.3 (1.2)	
	The forms I had to fill in for each patient are adapted and they correspond to the information I usually collect	6.8 (2.0)	
	Thanks to the export function provided in eDOL, I was able to retrieve the completed information for my patients. I was then able to print it (for my patient records) and/or import it into my hospital's electronic management system	5.0 (2.3)	
	The eDOL platform is complete enough to be able to replace my medical records one day	4.4 (1.9)	
	I would like to continue using eDOL in the future	7.3 (2.0)	
	eDOL will be useful in my daily medical practice	6.8 (1.6)	
	eDOL will allow me to better monitor my patients to improve their care	7.1 (1.6)	
	eDOL will be useful for developing clinical research on pain (creation of an e-cohort of patients with chronic pain)	9.0 (0.9)	
	eDOL will be useful for the clinical research projects conducted by my pain clinic	8.5 (1.7)	
**Patient^a^**			
	After reading the explanatory document provided by the physician, it was easy for me to use eDOL	8.4 (2.1)	
	After the first use, it is easy to use eDOL on a daily basis	8.7 (1.9)	
	The technical support was responsive enough when I asked for it	7.0 (2.7)	
	eDOL offers questionnaires and assessments that I feel are suitable for monitoring my pain and its impact on my daily life	7.0 (2.1)	
	I believe that the information I have entered in eDOL allows my doctor to better understand my pain and improve its management	6.9 (2.5)	
	During the time that I have been using eDOL, I feel that my doctor has better monitored my symptoms and that my pain has been better managed	5.7 (3.1)	
	I believe that the information I have entered in eDOL will also help researchers to better understand chronic pain and to identify new avenues of research	7.5 (2.3)	
	I think that eDOL will help me in my daily life to better manage my pain and its impact on my daily life	5.8 (2.7)	
	I think that eDOL will gradually improve my quality of life	5.6 (2.4)	
	I would like to continue using eDOL in the future	7.6 (2.8)	

^a^88.5% indicated they would participate in the next phase of study on the new version of eDOL.

### Secondary Objectives

We obtained 4 clusters that did not differ with respect to sociodemographic and chronic pain characteristics (except for pain interference with daily life) and their treatments (Multimedia Appendix 4). Interestingly, all patient characteristics obtained from validated biopsychosocial questionnaires differed between profiles. In particular, the patients in cluster 4 had more severe scores in various biopsychosocial and comorbidity scales (precariousness, anxiety, depression, kinesiophobia, sleep and cognitive disorders; *P*<.001) associated with a greater impact of pain and conversely for cluster 1. Clusters 2 and 3 were intermediate groups.

In Cluster 4, 80.0% (24/30) of patients adhered to the use of the tool, compared with 51.0% (19/37), 64.3% (9/14), and 43.5% (10/23) in clusters 1, 2, and 3, respectively (Multimedia Appendix 4). Moreover, type of pain was also related to adherence, with patients suffering from nociplastic pain who seemed to be more adherent than others (30/45, 66.7%; *P*=.01). It is noteworthy that 2 other items (presence of cognitive disorders and alexithymia) were related to adherence (*P*=.04), but with a small effect size (Cramer *V*=.03 and Cramer *V*=.20 respectively).

With reference to the profile of patients in cluster 4, the most severe patients, with a significant impact of pain on their daily life (*P*=.03), seemed to be those who adhered most to eDOL (24/62, 38.7% of patients who adhered to the use of the tool were in cluster 4).

## Discussion

### Overview

As this was primarily a feasibility study, we first discuss considerations regarding the data collection and acceptability, and then our exploratory results with respect to conducting future works and improving eDOL. Because of the low number of patients (and thus the limited longitudinal outcome data collected), we did not explore the impact of eDOL on pain disorders and related comorbidities.

### Feasibility

Our results showed a rate of adherence, after 3-month follow-up, of approximately 60% (65/105, 61.9%) of patients using eDOL. Three similar recent studies [24,27,31], which assessed a smartphone app that enables patients with chronic pain to assess, monitor, and communicate their status to their providers, showed that 76%, 70%, and 72% of patients used the app for 3 months. Another study [20], which assessed a remotely delivered pain management program in a web-based format (web platform), showed that 76% of patients adhered [20] for at least 3 months. A study [75] with adolescents with chronic pain showed a high level of adherence (78%) and satisfaction, and a study [72] with patients with multiple sclerosis and migraine that evaluated the feasibility of using a smartphone app for patient follow-up showed an adherence rate of 49% after 90 days. The eDOL tool seems to be accepted in a similar way to these other smartphone-based or web-based apps. In our study, only an email reminder was sent to our patients if they had not used the app within 2 weeks after their inclusion and only 1 visit (included in their usual care path) was scheduled after at least 3 months. The studies [20,24,27,31,72,75] cited above included regular telephone follow-up or frequent visits. Moreover, according to the mean score (5.7, SD 3.1) for the statement “During the time that I have been using eDOL, I feel that my doctor has better monitored my symptoms and that my pain has been better managed,” patients perceived that there was a lack of involvement of physicians in the eDOL tool. A study [76] showed that strong involvement by physicians increases adherence and the effectiveness of eHealth tools. Therefore, we can assume that a closer relationship with our patients (medical follow-up rhythm and involvement of physicians) would have further increased their adherence. This is undoubtedly a direction of research that should be taken for the future use of the app and patient follow-up; however, we must keep in mind that the aim of a real-life eHealth app is to be of little or no constraint for patients and to improve their medical follow-up, while lightening the physician's workload.

The good acceptability score, from both patients and physicians, reflects the interest expressed for eDOL and its contribution to the follow-up. Thus, eDOL could meet the urgent need to develop self-management and chronic pain management strategies through eHealth programs (internet, smartphone apps), and their therapeutic interest, as described by several studies [19,20,24-30].

### Exploratory Analyses

In our exploratory analyses, our study population was similar to the profile of patients suffering from chronic pain in France [77], Germany [78], the United Kingdom [2], Canada [79], or the United States [74,80,81]—predominantly female, middle-aged, active population of lower socioeconomic status (precariousness, employment status, level of education), with pain lasting more than 5 years and suffering from psychological distress and from fairly severe chronic pain that has a significant impact on their lives (92% with moderate-to-severe pain, 20% with high impact chronic pain [74] and 43% with sleep disorders, such as awakenings due to pain at least once a night), mainly treated by antidepressants, and weak opioids. Interestingly, most did not simultaneously explore sociodemographic, psychological, pain disorders, and treatments characteristics.

With our smartphone app, we were able to collect data on precariousness, kinesiophobia, catastrophism, alexithymia, feelings of injustice, personality, life satisfaction, beliefs about pain, anxiety-depression, sleep, quality of life, cognitive disorders, optimism and belief in a just world. We made this choice because all of these factors are related to chronic pain [38,40,50,77,82-90] and we wanted to evaluate the ability of eDOL to characterize our patients precisely. Thus, the strength of eDOL is that it enables the integration of a large panel of validated questionnaires that, in turn, enable the precise characterization of the patients, especially regarding their emotional and psychological state, chronic pain, and related comorbidities. This characterization will eventually provide a large amount of data for care and research, and rely on a multimodal exploratory analysis of the determinants and repercussions of chronic pain, and their evolution in a real-life context, taking into account all the environmental events likely to influence chronic pain (treatments, history, comorbidities).

Finally, the multifactorial analysis of all our data enabled us to group our study population into 4 clusters. Interestingly, subpopulations of our patients could be distinguished only on the basis of biopsychosocial questionnaires and impact of pain on daily life whereas sociodemographic aspects, symptomatology, seniority and treatment of pain did not differ between our clusters. Cluster 4 represented patients with more disabling chronic pain, more severe comorbidities, and more pronounced psychological disorders, while cluster 1 represented patients with chronic pain that has little impact on their daily life, as well as a lower presence of comorbidity. Cluster 4 had a higher proportion of adherent patients. Our findings were similar results to those in a recent study [31], which showed that adherent patients correspond to patients with high impact chronic pain. These results seem consistent because patients with high impact chronic pain [74] and associated comorbidities are more in need of a tool that potentially improves their medical follow-up and are therefore more inclined to use it. Moreover, nociplastic pain was related to adherence (*P*=.01). According to our experience with chronic pain treatment management, this characteristic could be explained both by the fact that patients suffering from nociplastic pain (especially fibromyalgia) are younger than the general chronic pain population (and thus, more digital friendly) and very involved in the management of their pain. Interestingly, the presence of cognitive disorders and alexithymia, independent of clusters, was related to adherence (*P*=.04). We hypothesize that patients with these disorders are aware of this and compensate by using eDOL as a digital companion, resulting in better adherence.

In addition, our results support the importance of questionnaires assessing the biopsychosocial aspect of chronic pain in addition to the biomedical aspect in the medical follow-up and characterization of patients with chronic pain. Moreover, in a classical medical follow-up, patients typically only see their pain specialist every 3 to 6 months. During these interviews, patients often have difficulties recalling their various symptoms and the impact of their pain over the past few months, which corresponds to a recall or memory bias [91]. Nevertheless, a review [92] demonstrates that the results of previous studies investigating this topic are highly variable. Some studies have shown that pain is remembered accurately [93-95], but others highlighted that patients tend to overestimate [96,97] or underestimate their pain [98]. Thus, a definitive answer to this question is still lacking, but real-life monitoring of different biopsychosocial and biomedical factors related to pain (not only pain intensity), using digital tools such as eDOL, could be a benefit in treatment management and the follow-up of patients.

### Limitations

There was a selection bias mainly because requiring the use of a smartphones excludes patients who do not have or do not know how to use this tool. This could exclude the older or more precarious patients. Nevertheless, in view of our results, the age of the participants and the rate of precariousness were similar to those found in the general French population, with and without chronic pain [77,99]. We also observed that our population included many patients with nociplastic pain (mainly fibromyalgia, 50/80, 62.5%), which was not the case in other foreign studies [20,24,27,31,72,75]. Another French study [77] also found a high rate of fibromyalgia (42%), which seems to show that the population of French pain clinics includes a large proportion of fibromyalgia patients. Thus, we can conclude that this bias has little impact on our results. The second limitation was a measurement bias, which occurs frequently in observational studies [100]. Nevertheless, self-reporting permits a wider range of responses than many other data collection designs [101]. Measurement bias can arise from recall period, selective recall, social desirability, or sampling approach. In our study, the recall period might be the major risk [100]. Since all the questions dealt with the present moment or, at the latest, 1 to 2 weeks earlier, the recall bias can be considered negligible.

Moreover, our satisfaction survey was not a standardized but was a custom-made tool. We built this tool based on existing tools, such as the Patient Satisfaction Questionnaire [69] and the Client Satisfaction Questionnaire [70,71], and adapted it to our study and to the eDOL tool so that we could have specific feedback for improvement. It should be noted that the tools on which ours were based have little or no relevance to mHealth interventions [70], hence the need to create one adapted specifically for our study.

Finally, only physicians were involved in this feasibility study; other members of the care team, such as nurses, physiotherapists, and psychologists, did not participate in the study. The absence of the point of view of the rest of care teams is a limitation to the interpretation of the acceptability of the eDOL tool. In future studies of the eDOL tool, we plan to include all the members of the care team as well as the addition of a chatbot and a new therapeutic education tool.

### Conclusions

The study demonstrated the feasibility and acceptability of eDOL for both patients with chronic pain and their physicians. These points justify continuing the deployment of the tool while providing information to improve its use and adherence to provide patients with chronic pain and their physicians with a better longitudinal characterization of pain and its impacts for an optimized and more personalized therapeutic management.

## Appendix

Multimedia Appendix 1 Patient and physician satisfaction questionnaires.

Multimedia Appendix 2 eDOL features.

Multimedia Appendix 3 Questionnaire completion data.

Multimedia Appendix 4 Study population details.

## Abbreviations

mHealth: mobile health

## References

[1] Elliott AM, Smith BH, Penny KI, Smith WC, Chambers WA (1999). The epidemiology of chronic pain in the community. Lancet.

[2] van Hecke O, Torrance N, Smith BH (2013). Chronic pain epidemiology and its clinical relevance. Br J Anaesth.

[3] Docking RE, Fleming J, Brayne C, Zhao J, Macfarlane GJ, Jones GT, Cambridge City over-75s Cohort Study collaboration (2011). Epidemiology of back pain in older adults: prevalence and risk factors for back pain onset. Rheumatology (Oxford).

[4] Thomas E, Peat G, Harris L, Wilkie R, Croft PR (2004). The prevalence of pain and pain interference in a general population of older adults: cross-sectional findings from the North Staffordshire Osteoarthritis Project (NorStOP). Pain.

[5] Gobina I, Villberg J, Välimaa R, Tynjälä J, Whitehead R, Cosma A, Brooks F, Cavallo F, Ng K, de Matos MG, Villerusa A (2019). Prevalence of self-reported chronic pain among adolescents: evidence from 42 countries and regions. Eur J Pain.

[6] Larsson C, Hansson EE, Sundquist K, Jakobsson U (2017). Chronic pain in older adults: prevalence, incidence, and risk factors. Scand J Rheumatol.

[7] GBD 2016 DiseaseInjury IncidencePrevalence Collaborators (2017). Global, regional, and national incidence, prevalence, and years lived with disability for 328 diseases and injuries for 195 countries, 1990-2016: a systematic analysis for the Global Burden of Disease Study 2016. Lancet.

[8] Breivik H, Collett B, Ventafridda V, Cohen R, Gallacher D (2006). Survey of chronic pain in Europe: prevalence, impact on daily life, and treatment. Eur J Pain.

[9] Attal N, Lanteri-Minet M, Laurent B, Fermanian J, Bouhassira D (2011). The specific disease burden of neuropathic pain: results of a French nationwide survey. Pain.

[10] Impact of pain on society costs the EU up to 441 billion Euros annually. Societal Impact of Pain (SIP).

[11] Gaskin DJ, Richard P (2012). The economic costs of pain in the United States. J Pain.

[12] Breivik H, Eisenberg E, O'Brien T, OPENMinds (2013). The individual and societal burden of chronic pain in Europe: the case for strategic prioritisation and action to improve knowledge and availability of appropriate care. BMC Public Health.

[13] Global medicine spending and usage trends: outlook to 2025. The IQVIA Institute.

[14] The IQVIA Institute.

[15] Crofford LJ (2010). Adverse effects of chronic opioid therapy for chronic musculoskeletal pain. Nat Rev Rheumatol.

[16] Mogil JS (2009). Animal models of pain: progress and challenges. Nat Rev Neurosci.

[17] Mouraux André, Bannister Kirsty, Becker Susanne, Finn David P, Pickering Gisèle, Pogatzki-Zahn Esther, Graven-Nielsen Thomas (2021). Challenges and opportunities in translational pain research - an opinion paper of the Working Group on Translational Pain Research of the European Pain Federation (EFIC). Eur J Pain.

[18] Bouhassira D, Branders S, Attal N, Fernandes AM, Demolle D, Barbour J, Ciampi de Andrade D, Pereira A (2021). Stratification of patients based on the neuropathic pain symptom inventory: development and validation of a new algorithm. Pain.

[19] Jamison RN, Jurcik DC, Edwards RR, Huang C, Ross EL (2017). A pilot comparison of a smartphone app with or without 2-way messaging among chronic pain patients: who benefits from a pain app?. Clin J Pain.

[20] Manjula BN, Acharya AS, Vithayathil PJ (1976). Deamidated active intermediates in the irreversible acid denaturation of ribonuclease-A. Int J Pept Protein Res.

[21] Suso-Ribera C, Castilla D, Zaragozá I, Ribera-Canudas MV, Botella C, García-Palacios A (2018). Validity, reliability, feasibility, and usefulness of pain monitor: a multidimensional smartphone app for daily monitoring of adults with heterogenous chronic pain. Clin J Pain.

[22] Shadd JD, Ryan BL, Maddocks HL, McKay SD, Moulin DE (2015). Neuropathic pain in a primary care electronic health record database. Eur J Pain.

[23] Minen MT, Jalloh A, Ortega E, Powers SW, Sevick MA, Lipton RB (2019). User design and experience preferences in a novel smartphone application for migraine management: a think aloud study of the RELAXaHEAD application. Pain Med.

[24] Jamison RN, Mei A, Ross EL (2018). Longitudinal trial of a smartphone pain application for chronic pain patients: predictors of compliance and satisfaction. J Telemed Telecare.

[25] Gogovor A, Visca R, Auger C, Bouvrette-Leblanc L, Symeonidis I, Poissant L, Ware MA, Shir Y, Viens N, Ahmed S (2017). Informing the development of an internet-based chronic pain self-management program. Int J Med Inform.

[26] McGuire BE, Henderson EM, McGrath PJ (2017). Translating e-pain research into patient care. Pain.

[27] Sundararaman LV, Edwards RR, Ross EL, Jamison RN (2017). Integration of mobile health technology in the treatment of chronic pain. Reg Anesth Pain Med.

[28] Martin CL, Bakker CJ, Breth MS, Gao G, Lee K, Lee MA, Tiase VL, Tunby LJ, Wyatt TH, Janeway LM (2021). The efficacy of mobile health interventions used to manage acute or chronic pain: a systematic review. Res Nurs Health.

[29] Suso-Ribera C, Castilla D, Zaragozá I, Server A, Medel J, García-Palacios A, Mesas (2020). Telemonitoring in chronic pain management using smartphone apps: a randomized controlled trial comparing usual assessment against app-based monitoring with and without clinical alarms. Int J Environ Res Public Health.

[30] Mariano TY, Wan L, Edwards RR, Lazaridou A, Ross EL, Jamison RN (2021). Online group pain management for chronic pain: preliminary results of a novel treatment approach to teletherapy. J Telemed Telecare.

[31] Ross EL, Jamison RN, Nicholls L, Perry BM, Nolen KD (2020). Clinical integration of a smartphone app for patients with chronic pain: retrospective analysis of predictors of benefits and patient engagement between clinic visits. J Med Internet Res.

[32] Dear BF, Courtney C, Khor KE, McDonald S, Ricciardi T, Gandy M, Fogliati VJ, Titov N (2018). The pain course: exploring the feasibility of an internet-delivered pain management program when offered by a tertiary pain management service. Clin J Pain.

[33] Dear BF, Gandy M, Karin E, Fogliati R, Fogliati VJ, Staples LG, Wootton BM, Sharpe L, Titov N (2018). The pain course: 12- and 24-month outcomes from a randomized controlled trial of an internet-delivered pain management program provided with different levels of clinician support. J Pain.

[34] Dear BF, Gandy M, Karin E, Staples LG, Johnston L, Fogliati VJ, Wootton BM, Terides MD, Kayrouz R, Perry KN, Sharpe L, Nicholas MK, Titov N (2015). The pain course: a randomised controlled trial examining an internet-delivered pain management program when provided with different levels of clinician support. Pain.

[35] World Medical Association (2013). World Medical Association Declaration of Helsinki: ethical principles for medical research involving human subjects. JAMA.

[36] Code de la santé publique article R5121-13. Légifrance.

[37] Code pénal article 226-13. Légifrance.

[38] Williams DA, Thorn BE (1989). An empirical assessment of pain beliefs. Pain.

[39] Sass C, Moulin JJ, Gueguen R, Abric L, Dauphinot V, Dupre C, Giordanella J P, Girard F, Guenot C, Labbe E, La Rosa E, Magnier P, Martin E, Royer B, Rubirola M, Gerbaud L (2006). Le score Epices?: un score individuel de précarité construction du score et mesure des relations avec des données de santé, dans une population de 197 389 personnes. Bulletin Epidémiologique Hebdomadaire.

[40] Sullivan MJL, Adams H, Horan S, Maher D, Boland D, Gross R (2008). The role of perceived injustice in the experience of chronic pain and disability: scale development and validation. J Occup Rehabil.

[41] Maslach C, Jackson SE (1981). The measurement of experienced burnout. J Organiz Behav.

[42] Bagby RM, Taylor GJ, Parker JD (1994). The twenty-item Toronto Alexithymia Scale--II. convergent, discriminant, and concurrent validity. J Psychosom Res.

[43] Scheier MF, Carver CS, Bridges MW (1994). Distinguishing optimism from neuroticism (and trait anxiety, self-mastery, and self-esteem): a reevaluation of the Life Orientation Test. J Pers Soc Psychol.

[44] Lucas T, Zhdanova L, Alexander S (2011). Procedural and distributive justice beliefs for self and others. J Individ Differ.

[45] Karasek R, Brisson C, Kawakami N, Houtman I, Bongers P, Amick B (1998). The Job Content Questionnaire (JCQ): an instrument for internationally comparative assessments of psychosocial job characteristics. J Occup Health Psychol.

[46] John O, Srivastava S (1999). The Big Five Trait taxonomy: history, measurement, and theoretical perspectives. Handbook of Personality: Theory and Research 2nd ed.

[47] Cleeland CS, Ryan KM (1994). Pain assessment: global use of the brief pain inventory. Ann Acad Med Singapore.

[48] Hays RD, Martin SA, Sesti AM, Spritzer KL (2005). Psychometric properties of the Medical Outcomes Study sleep measure. Sleep Med.

[49] Miller RP, Kori SH, Todd DD (1991). The Tampa scale. Clin J Pain.

[50] Sullivan M, Stanish W, Waite H, Sullivan M, Tripp D (1998). Catastrophizing, pain, and disability in patients with soft-tissue injuries. Pain.

[51] Waddell G, Newton M, Henderson I, Somerville D, Main CJ (1993). A fear-avoidance beliefs questionnaire (FABQ) and the role of fear-avoidance beliefs in chronic low back pain and disability. Pain.

[52] EQ-5D-3L. EuroQol.

[53] Zigmond AS, Snaith RP (1983). The hospital anxiety and depression scale. Acta Psychiatr Scand.

[54] Diener E, Emmons RA, Larsen RJ, Griffin S (1985). The satisfaction with life scale. J Pers Assess.

[55] Thomas Antérion C, Ribas C, Honoré-Masson S, Million J, Laurent B (2004). Evaluation de la plainte cognitive de patients Alzheimer, de sujets MCI, anxiodépressifs et de témoins avec le QPC (Questionnaire de Plainte Cognitive). Neurologie - Psychiatrie - Gériatrie.

[56] Bouhassira D, Attal N, Fermanian J, Alchaar H, Gautron M, Masquelier E, Rostaing S, Lanteri-Minet M, Collin E, Grisart J, Boureau F (2004). Development and validation of the neuropathic pain symptom inventory. Pain.

[57] Bellamy N, Buchanan WW, Goldsmith CH, Campbell J, Stitt LW (1988). Validation study of WOMAC: a health status instrument for measuring clinically important patient relevant outcomes to antirheumatic drug therapy in patients with osteoarthritis of the hip or knee. J Rheumatol.

[58] Gossec L, Paternotte S, Aanerud GJ, Balanescu A, Boumpas DT, Carmona L, de Wit M, Dijkmans BAC, Dougados M, Englbrecht M, Gogus F, Heiberg T, Hernandez C, Kirwan JR, Mola EM, Cerinic MM, Otsa K, Schett G, Scholte-Voshaar M, Sokka T, von Krause G, Wells GA, Kvien TK (2011). Finalisation and validation of the rheumatoid arthritis impact of disease score, a patient-derived composite measure of impact of rheumatoid arthritis: a EULAR initiative. Ann Rheum Dis.

[59] Roland M, Morris R (1983). A study of the natural history of back pain. part I: development of a reliable and sensitive measure of disability in low-back pain. Spine (Phila Pa 1976).

[60] Francis CY, Morris J, Whorwell PJ (1997). The irritable bowel severity scoring system: a simple method of monitoring irritable bowel syndrome and its progress. Aliment Pharmacol Ther.

[61] Burckhardt CS, Clark SR, Bennett RM (1991). The fibromyalgia impact questionnaire: development and validation. J Rheumatol.

[62] Kosinski M, Bayliss MS, Bjorner JB, Ware JE, Garber WH, Batenhorst A, Cady R, Dahlöf CGH, Dowson A, Tepper S (2003). A six-item short-form survey for measuring headache impact: the HIT-6. Qual Life Res.

[63] Hurst H, Bolton J (2004). Assessing the clinical significance of change scores recorded on subjective outcome measures. J Manipulative Physiol Ther.

[64] Blanchard EB, Jones-Alexander J, Buckley TC, Forneris CA (1996). Psychometric properties of the PTSD checklist (PCL). Behav Res Ther.

[65] Bouhassira D, Attal N, Alchaar H, Boureau F, Brochet B, Bruxelle J, Cunin G, Fermanian J, Ginies P, Grun-Overdyking A, Jafari-Schluep H, Lantéri-Minet M, Laurent B, Mick G, Serrie A, Valade D, Vicaut E (2005). Comparison of pain syndromes associated with nervous or somatic lesions and development of a new neuropathic pain diagnostic questionnaire (DN4). Pain.

[66] Perrot S, Bouhassira D, Fermanian J, CEDR (Cercle d'Etude de la Douleur en Rhumatologie) (2010). Development and validation of the Fibromyalgia Rapid Screening Tool (FiRST). Pain.

[67] Knisely JS, Wunsch MJ, Cropsey KL, Campbell ED (2008). Prescription opioid misuse index: a brief questionnaire to assess misuse. J Subst Abuse Treat.

[68] Webster LR, Webster RM (2005). Predicting aberrant behaviors in opioid-treated patients: preliminary validation of the opioid risk tool. Pain Med.

[69] Thayaparan AJ, Mahdi E (2013). The patient satisfaction questionnaire short form (PSQ-18) as an adaptable, reliable, and validated tool for use in various settings. Med Educ Online.

[70] Boß L, Lehr D, Reis D, Vis C, Riper H, Berking M, Ebert DD (2016). Reliability and validity of assessing user satisfaction with web-based health interventions. J Med Internet Res.

[71] Larsen DL, Attkisson CC, Hargreaves WA, Nguyen TD (1979). Assessment of client/patient satisfaction: development of a general scale. Eval Program Plann.

[72] Minen MT, Schaubhut KB, Morio K (2020). Smartphone based behavioral therapy for pain in multiple sclerosis (MS) patients: A feasibility acceptability randomized controlled study for the treatment of comorbid migraine and ms pain. Mult Scler Relat Disord.

[73] Ward JH (1963). Hierarchical grouping to optimize an objective function. J Am Stat Assoc.

[74] Von Korff M, Scher AI, Helmick C, Carter-Pokras O, Dodick DW, Goulet J, Hamill-Ruth R, LeResche L, Porter L, Tait R, Terman G, Veasley C, Mackey S (2016). United States national pain strategy for population research: concepts, definitions, and pilot data. J Pain.

[75] Shaygan M, Jaberi A (2021). The effect of a smartphone-based pain management application on pain intensity and quality of life in adolescents with chronic pain. Sci Rep.

[76] Mariano TY, Wan L, Edwards RR, Jamison RN (2019). Online teletherapy for chronic pain: a systematic review. J Telemed Telecare.

[77] Dany L, Roussel P, Laguette V, Lagouanelle-Simeoni M, Apostolidis T (2016). Time perspective, socioeconomic status, and psychological distress in chronic pain patients. Psychol Health Med.

[78] Frettlöh J, Maier C, Gockel H, Zenz M, Hüppe M (2009). [Characterization of chronic pain patients in German pain centers : core data from more than 10,000 patients]. Schmerz.

[79] May C, Brcic V, Lau B (2018). Characteristics and complexity of chronic pain patients referred to a community-based multidisciplinary chronic pain clinic. Can J Pain.

[80] Malon J, Shah P, Koh WY, Cattabriga G, Li E, Cao L (2018). Characterizing the demographics of chronic pain patients in the state of Maine using the Maine all payer claims database. BMC Public Health.

[81] Dahlhamer J, Lucas J, Zelaya Carla, Nahin R, Mackey S, DeBar L, Kerns R, Von Korff M, Porter L, Helmick C (2018). Prevalence of chronic pain and high-impact chronic pain among adults - United States, 2016. MMWR Morb Mortal Wkly Rep.

[82] Luque-Suarez A, Martinez-Calderon J, Falla D (2019). Role of kinesiophobia on pain, disability and quality of life in people suffering from chronic musculoskeletal pain: a systematic review. Br J Sports Med.

[83] Di Tella M, Castelli L (2016). Alexithymia in chronic pain disorders. Curr Rheumatol Rep.

[84] Ibrahim ME, Weber K, Courvoisier DS, Genevay S (2020). Big five personality traits and disabling chronic low back pain: association with fear-avoidance, anxious and depressive moods. J Pain Res.

[85] Stålnacke B (2011). Life satisfaction in patients with chronic pain - relation to pain intensity, disability, and psychological factors. Neuropsychiatr Dis Treat.

[86] Emery PC, Wilson KG, Kowal J (2014). Major depressive disorder and sleep disturbance in patients with chronic pain. Pain Res Manag.

[87] Mutubuki EN, Beljon Y, Maas ET, Huygen FJPM, Ostelo RWJG, van Tulder MW, van Dongen JM (2020). The longitudinal relationships between pain severity and disability versus health-related quality of life and costs among chronic low back pain patients. Qual Life Res.

[88] Baker KS, Gibson SJ, Georgiou-Karistianis N, Giummarra MJ (2018). Relationship between self-reported cognitive difficulties, objective neuropsychological test performance and psychological distress in chronic pain. Eur J Pain.

[89] Bargiel-Matusiewicz K, Krzyszkowska A (2009). Dispositional optimism and coping with pain. Eur J Med Res.

[90] McPartland JM, Matias I, Di Marzo V, Glass M (2006). Evolutionary origins of the endocannabinoid system. Gene.

[91] Heron KE, Smyth JM (2010). Ecological momentary interventions: incorporating mobile technology into psychosocial and health behaviour treatments. Br J Health Psychol.

[92] Schoth DE, Radhakrishnan K, Liossi C (2020). A systematic review with subset meta-analysis of studies exploring memory recall biases for pain-related information in adults with chronic pain. Pain Rep.

[93] Bąbel P (2017). The effect of positive affect on the memory of pain. Pain Manag Nurs.

[94] Jamison RN, Raymond SA, Slawsby EA, McHugo GJ, Baird JC (2006). Pain assessment in patients with low back pain: comparison of weekly recall and momentary electronic data. J Pain.

[95] Hovasapian A, Levine LJ (2016). Reappraisal mitigates overestimation of remembered pain in anxious individuals. Cogn Emot.

[96] Broderick JE, Schwartz JE, Vikingstad G, Pribbernow M, Grossman S, Stone AA (2008). The accuracy of pain and fatigue items across different reporting periods. Pain.

[97] Gedney JJ, Logan H (2006). Pain related recall predicts future pain report. Pain.

[98] Rode S, Salkovskis PM, Jack T (2001). An experimental study of attention, labelling and memory in people suffering from chronic pain. Pain.

[99] Labbé É, Moulin J J, Guéguen R, Sass C, Chatain C, Gerbaud L (2007). Un indicateur de mesure de la précarité et de la « santé sociale » : le score EPICES: L'expérience des Centres d'examens de santé de l'Assurance maladie. La Revue de l'Ires.

[100] Althubaiti A (2016). Information bias in health research: definition, pitfalls, and adjustment methods. J Multidiscip Healthc.

[101] Zhu K, McKnight B, Stergachis A, Daling JR, Levine RS (1999). Comparison of self-report data and medical records data: results from a case-control study on prostate cancer. Int J Epidemiol.

