# Supplementary material for: eDOL mHealth App and Web Platform for Self-monitoring and Medical Follow-up of Patients With Chronic Pain: Observational Feasibility Study
Source: JMIR Form Res. 2022 Mar 2;6(3):e30052. doi: 10.2196/30052 (PMC8928045; doi:10.2196/30052)
Supplement: Multimedia Appendix 1 [file formative_v6i3e30052_app1.doc]

**TABLE 1 – PHYSICIAN AND PATIENT ACCEPTABILITY OF eDOL**

| **Physician acceptability questionnaire** | **Score** (/10 ±SD) |  |
| --- | --- | --- |
| 1) The training and support provided was sufficient to use eDOL correctly | 7.3±1.4 |  |
| 2) After the first training session, it is easy to use eDOL on a daily basis | 6.9±2.3 |  |
| 3) The technical support (email and phone) was available to assist me if needed | 8.3±1.2 |  |
| 4) eDOL offers questionnaires and meters adapted to the multidimensional characterization of my patients | 8.3±1.2 |  |
| 5) The forms I had to fill in for each patient are adapted and they correspond to the information I usually collect | 6.8±2.0 |  |
| 6) Thanks to the export function provided in eDOL, I was able to retrieve the completed information for my patients. I was then able to print it (for my patient records) and/or import it into my hospital's electronic management system | 5.0±2.3 |  |
| 7) The eDOL platform is complete enough to be able to replace my medical records one day | 4.4±1.9 |  |
| 8) I would like to continue using eDOL in the future | 7.3±2.0 |  |
| 9) eDOL will be useful in my daily medical practice | 6.8±1.6 |  |
| 10) eDOL will allow me to better monitor my patients to improve their care | 7.1±1.6 |  |
| 11) eDOL will be useful for developing clinical research on pain (creation of an e-cohort of chronic pain patients) | 9.0±0.9 |  |
| 12) eDOL will be useful for the clinical research projects conducted by my pain clinic | 8.5±1.7 |  |
| **Patient acceptability questionnaire** | **Score** (/10 ±SD) |  |
| 1) After reading the explanatory document provided by the physician, it was easy for me to use eDOL | 8.4±2.1 |  |
| 2) After the first use, it is easy to use eDOL on a daily basis | 8.7±1.9 |  |
| 3) The technical support was responsive enough when I asked for it | 7.0±2.7 |  |
| 4) eDOL offers questionnaires and meters that I feel are suitable for monitoring my pain and its impact on my daily life | 7.0±2.1 |  |
| 5) I believe that the information I have entered in eDOL allows my doctor to better understand my pain and improve its management | 6.9±2.5 |  |
| 6) During the time that I have been using eDOL, I feel that my doctor has better monitored my symptoms and that my pain has been better managed | 5.7±3.1 |  |
| 7) I believe that the information I have entered in eDOL will also help researchers to better understand chronic pain and to identify new avenues of research | 7.5±2.3 |  |
| 8) I think that eDOL will help me in my daily life to better manage my pain and its impact on my daily life | 5.8±2.7 |  |
| 9) I think that eDOL will gradually improve my quality of life | 5.6±2.4 |  |
| 10) I would like to continue using eDOL in the future | 7.6±2.8 |  |
| 11) I would like to participate in the next phase of experimentation on the new version of eDOL | 88.5% YES |  |
