# Supplementary material for: eDOL mHealth App and Web Platform for Self-monitoring and Medical Follow-up of Patients With Chronic Pain: Observational Feasibility Study
Source: JMIR Form Res. 2022 Mar 2;6(3):e30052. doi: 10.2196/30052 (PMC8928045; doi:10.2196/30052)
Supplement: Multimedia Appendix 2 [file formative_v6i3e30052_app2.doc]

**TABLE 2** – LIST OF eDOL FEATURES

| **eDOL V1.0** | **Smartphone Application** | **Investigator web platform** | **Repeatability** | **Details** |
| --- | --- | --- | --- | --- |
| **Inclusion form** |  | **✓** | **NA** | **Last name / first name / email / anonymity number** |
|  |  |  |  | **History** (clinical / psy / drug) **/ clinical examination / medico-economic aspect** (the different consultations of the patient) / diagnosis of pain according to **ICD11 classification.** |
| **Personal information** | **✓** |  | **NA** | **Socio-demographic elements:** work / alcohol / tobacco |
|  |  |  |  | **Pain characterization:** frequency / duration / aggravating and alleviating factors, etc. |
| **Treatments form** |  | **✓** | Updated at each consultation | **Analgesics:** name / dates / dosage / side effects. List of non-medicinal techniques + other treatments (free text). |
| **Meters** | **✓** |  | Weekly | **11-point NRS (0-10):** sleep / morale / fatigue & energy / body comfort / anxiety / pain |
| **Self-questionnaires** | **✓** |  | **not repeated** or every **3** or **6-months** | **5 sessions of questionnaires spread over the first 2 weeks post-inclusion:** |
|  |  |  |  | **Not repeated:** FABQ^a^ / IEQ MBI^a^ / PBPI / EPICES / JCQ^a^ / LOT-R / BJW / PCLS^b^ / TAS-20 BFI |
|  |  |  |  | **Every 3 months:** FIQ^c^ / HIT-6^c^ / IBS-SSS^c^ / POMI^b^ / PGIC^b^ / NPSI^b^ / RAID^b^ / BPI / MOS Sleep Scale |
|  |  |  |  | **Every 6 months:** TSK / RMDQ^c^ / WOMAC^c^ / PCS / EQ-5D-3L / HADS / SWLS / SCC |
| **Hetero-questionnaires** |  | **✓** | **NA** | **Diagnostic validation**: DN4 + NEUPSIG (neuropathy) / WPI/SSS + FIRST (fibromyalgia) / ROME IV (irritable bowel syndrome) |
|  |  |  |  | **Others**: ORT (updated at each consultation) |
| **Consultation form** |  | **✓** | Updated at each consultation | **clinical examination / medico-eco aspect / observance / benefit-risk ratio of treatments** |
| *^a^ work-related questionnaires; ^b^ optional questionnaires; ^c^ disease-specific questionnaires* | | | | |
