# Supplementary material for: eDOL mHealth App and Web Platform for Self-monitoring and Medical Follow-up of Patients With Chronic Pain: Observational Feasibility Study
Source: JMIR Form Res. 2022 Mar 2;6(3):e30052. doi: 10.2196/30052 (PMC8928045; doi:10.2196/30052)
Supplement: Multimedia Appendix 3 [file formative_v6i3e30052_app3.doc]

**TABLE 3 - PERCENTAGE OF FILLING IN THE DIFFERENT QUESTIONNAIRES / METER**S / FORMS

| **Physician baseline and follow-up forms** | **Patients with completed form** *n=105*  n, (%) |  | |
| --- | --- | --- | --- |
| Inclusion form *(baseline)* | 77 (73.3) |  | |
| Diagnosis form *(baseline)* | 80 (76.2) |  | |
| Treatment form *(baseline and follow-up)* | 74 (70.5) |  | |
| Consultation form *(follow-up)* | 66 (62.9) |  | |
|  |  |  | |
| **Self-administered quest. and meters** | **Patients with baseline filling** *n=105*  n, (%) | **Patient with repeated filling** (n, %) | |
|  |  | **3-months follow-up** *n=105* | **6-months follow-up** *n=65* |
| Weekly meters | 93 (88.6) | 65 (61.9) | 50 (76.9) |
| TAS-20 | 100 (95.2) | NA | NA |
| IEQ | 100 (95.2) | NA | NA |
| PBPI | 92 (87.6) | NA | NA |
| LOT-R | 94 (89.5) | NA | NA |
| BJW | 94 (89.5) | NA | NA |
| EPICES | 93 (88.6) | NA | NA |
| BFI | 92 (87.6) | NA | NA |
| MOS-Sleep Scale | 94 (89.5) | 67 (63.8) | 39 (60.0) |
| BPI | 93 (88.6) | 67 (63.8) | 38 (58.5) |
| PCS | 100 (95.2) | NA | 40 (61.5) |
| SWLS | 92 (87.6) | NA | 35 (53.8) |
| SCC | 93 (88.6) | NA | 35 (53.8) |
| EQ-5D-3L | 83 (79.0) | NA | 36 (55.4) |
| HADS | 94 (89.5) | NA | 41 (63.1) |
| TSK | 93 (88.6) | NA | 41 (63.1) |
| *NA: not applicable* |  |  |  |
