# Supplementary material for: eDOL mHealth App and Web Platform for Self-monitoring and Medical Follow-up of Patients With Chronic Pain: Observational Feasibility Study
Source: JMIR Form Res. 2022 Mar 2;6(3):e30052. doi: 10.2196/30052 (PMC8928045; doi:10.2196/30052)
Supplement: Multimedia Appendix 4 [file formative_v6i3e30052_app4.doc]

**Multimedia Appendix 4.** Study population details.

|  | **Population** | |  |
| --- | --- | --- | --- |
|  | **Baseline** *n=105* | **3-months follow-up** *n=65* | ***p-value*** |
| **BASELINE SOCIO-DEMOGRAPHIC CHARACTERIZATION** | | | |
| **Female gender**, n (%) | 81 (77.1) | 51 (78.5) | .99 |
| **Age** (years), med [interquartile range] | 47.0 [38.0;55.0] | 47.0 [39.0;56.0] | .80 |
| *18 to 39 years, n (%)* | 30 (28.6) | 17 (26.2) | .87 |
| *40 to 59 years, n (%)* | 58 (55.2) | 38 (58.5) | .80 |
| *≥ 60 years, n (%)* | 17 (16.2) | 10 (15.4) | 1.0 |
| **Single***, n (%)* | 29 (27.6) | 20 (30.8) | .79 |
| **No children**, n (%) | 49 (46.7) | 29 (44.6) | .87 |
| **Level of education***, n (%)* |  |  |  |
| *A level or less* | 52 (49.5) | 35 (53.8) | .70 |
| *Diploma of Higher Education and BA, BS/BSc* | 33 (31.4) | 19 (29.2) | .90 |
| *MS/MSc, MA or more* | 20 (19.1) | 11 (17.0) | .88 |
| **Profession***, n (%)* |  |  |  |
| *Craftsmen, merchants, entrepreneurs, executives and senior professionals* | 19 (18.1) | 12 (18.4) | 1.0 |
| *Employees, Factory workers and Intermediate professions* | 50 (47.6) | 33 (50.8) | .81 |
| *Without professional activity* | 36 (34.3) | 20 (30.8) | .76 |
| **Professional situation***, n (%)* |  |  |  |
| *In activity* | 85 (80.9) | 55 (84.6) | .69 |
| *Work stoppage due to pain* | 30/85 (35.3) | 19/55 (34.5) | 1.0 |
| *Disability or Disabled adult allowance* | 27/85 (31.8) | 19/55 (34.5) | .87 |
| *Unemployed* | 15 (14.3) | 8 (12.3) | .89 |
| *Student* | 1 (1.0) | 0 (0.0) | 1.0 |
| *Retired* | 4 (3.8) | 2 (3.1) | 1.0 |
| **Tobacco***, n (%)* | 27 (25.7) | 15 (23.1) | .84 |
| **Alcohol***, n (%)* | 62 (59.0) | 35 (53.8) | .61 |
| **BASELINE QUESTIONNARIES SCORES** | | | |
| **Total score EPICES**, med [interquartile range] | 24.0 [14.0;41.0] | 24.0 [8.0;38.0] | .81 |
| *% precarious (score ≥30), n (%)* | 40/93 (43.0) | 27 (41.5) | .98 |
| **Total score TSK** (/68), med [interquartile range] | 43.0 [36.0;49.0] | 44.0 [36.0;50.0] | .56 |
| *% score ≥37 (proven kinesiophobia), n (%)* | 67/93 (72.0) | 48 (73.8) | .94 |
| **Total score IEQ** (/48), med [interquartile range] | 29.5 [19.8;36.3] | 30.0 [21.0;37.0] | .82 |
| **Total score TAS20** (/100), med [interquartile range] | 61.0 [53.0;68.0] | 63.0 [54.0;70.0] | .34 |
| *% score ≥61 (proven alexithymia), n (%)* | 51/100 (51.0) | 39 (60.0) | .33 |
| **BFI-44** (score /5), med [interquartile range] |  |  |  |
| *Extraversion* | 2.6 [2.2;3.1] | 2.6 [2.2;3.0] | .93 |
| *Agreeableness* | 4.2 [3.8;4.5] | 4.2 [3.8;4.6] | .65 |
| *Conscientiousness* | 3.7 [3.3;4.0] | 3.7 [3.3;4.0] | .99 |
| *Neuroticism* | 2.7 [2.1;3.1] | 2.8 [2.1;3.1] | .92 |
| *Openness to experience* | 3.7 [3.1;4.1] | 3.6 [3.1;4.0] | .66 |
| **MOS-Sleep Scale** |  |  |  |
| *% optimal sleep (7-8h / night), n (%)* | 31/94 (33.0) | 21 (32.3) | 1.0 |
| *Sleep Problem Index (SPI),* med [interquartile range] | 56.0 [42.0;69.0] | 56.0 [40.0;69.0] | .88 |
| **HADS** |  |  |  |
| *Total score anxiety,* med [interquartile range] | 9.0 [6.3;12.0] | 9.0 [7.0;12.0] | .98 |
| No symptom (score <7), n (%) | 24/94 (25.6) | 16 (24.6) | 1.0 |
| Borderline (score ≥7 to <11), n (%) | 35/94 (37.2) | 26 (40.0) | .85 |
| Clinical caseness (score ≥11), n (%) | 35/94 (37.2) | 23 (35.4) | .94 |
| *Total score depression,* med [interquartile range] | 8.0 [4.3;11.0] | 8.0 [4.0;11.0] | .87 |
| No symptom (score <7), n (%) | 36/94 (38.3) | 24 (36.9) | .99 |
| Borderline (score ≥7 to <11), n (%) | 32/94 (34.0) | 22 (33.8) | 1.0 |
| Clinical caseness (score ≥11), n (%) | 26/94 (27.7) | 19 (29.2) | .97 |
| **PBPI** (score -2 to +2), med [interquartile range] |  |  |  |
| *Mystery* | -0.20 [-1.00;0.20] | -0.20 [-1.00;0.20] | .98 |
| *Chronicity* | 0.67 [-0.33;1.33] | 0.67 [-0.33;1.00] | .68 |
| *Constancy* | 0.00 [-0.31;0.25] | 0.00 [-0.25;0.50] | .73 |
| *Self-Blame* | 0.67 [0.33;1.00] | 0.67 [0.33;1.00] | .97 |
| **Total score SWLS** (/35), med [interquartile range] | 18.5 [14.8;23.3] | 19.0 [14.8;24.3] | .86 |
| *% score ≤19 (degraded life satisfaction)*, n (%) | 51/92 (55.4) | 36 (55.4) | 1.0 |
| **Health status - EQ-5D-3L** (/100), med [interquartile range] | 51.0 [40.0;65.0] | 51.0 [40.0;65.5] | .90 |
| **Total score LOT-R** (/24), med [interquartile range] | 12.0 [10.3;13.0] | 12.0 [10.0;13.0] | .89 |
| **Total score BJW** (/30), med [interquartile range] | 17.0 [14.0;19.8] | 18.0 [14.0;20.0] | .82 |
| **Total score PCS** (/52), med [interquartile range] | 28.5 [20.0;36.3] | 29.0 [20.0;37.0] | .95 |
| *% score ≥30 (proven catastrophism)*, n (%) | 47/100 (47.0) | 32 (49.2) | .90 |
| **SCC,** *possible cognitive disorders*, n (%) | 77/93 (82.8) | 56 (86.2) | .73 |
| **BASELINE PAIN DISORDERS & TREATMENTS CHARACTERIZATION** | | | |
| **BPI** |  |  |  |
| *General pain intensity (/10),* med [interquartile range] | 6.0 [5.0;7.0] | 6.0 [5.0;7.0] | .98 |
| *<4/10 (mild pain), n (%)* | 7/83 (8.3) | 3/60 (5.0) | .52 |
| *≥4/10 and <7/10 (moderate pain), n (%)* | 36/83 (43.4) | 29/60 (48.3) | .68 |
| *≥7/10 (severe pain), n (%)* | 40/83 (48.2) | 28/60 (46.7) | .99 |
| *Total score pain interference (/10),* med [interquartile range] | 4.1 [3.3;4.9] | 4.0 [3.3;4.8] | .88 |
| *High impact chronic pain*, n (%)* | 17/83 (20.5) | 16/60 (26.7) | .51 |
| **Type of pain***, n (%)* |  |  |  |
| *Neuropathic (MG30.50/51 and others)* | 21/80 (26.3) | 10/44 (22.7) | .76 |
| *Nociceptive (MG30.20/21)* | 10/80 (12.5) | 3/44 (6.8) | .54 |
| *Nociplastic (MG30.00/01/02/42 and 8A80.1/2)* | 50/80 (62.5) | 31/44 (70.4) | .27 |
| *Others* | 10/80 (12.5) | 4/44 (9.1) | .77 |
| **Pain seniority***, n (%)* |  |  |  |
| *≤3 years* | 25 (23.8) | 18 (27.7) | .70 |
| *4-5 years* | 25 (23.8) | 15 (23.1) | .77 |
| *>5 years* | 55 (52.4) | 32 (49.2) | .81 |
| **Pain frequency***, n (%)* |  |  |  |
| *Continuous* | 56 (53.3) | 39 (60.0) | .49 |
| *Every day of the year* | 40 (38.1) | 22 (33.8) | .69 |
| *Several times a week* | 7 (6.7) | 3 (4.6) | .74 |
| *≤1 once a week* | 2 (1.9) | 1 (1.5) | 1.0 |
| **Pain paroxysms***, n (%)* |  |  |  |
| *Several times a day* | 30 (28.6) | 17(26.2) | .87 |
| *Every day of the year* | 41 (39.0) | 29(44.6) | .58 |
| *Rarely* | 33 (31.4) | 18(27.7) | .73 |
| *Never* | 1 (1.0) | 1 (1.5) | 1.0 |
| **Duration of pain paroxysms***, n (%)* |  |  |  |
| *> 2h* | 68 (64.8) | 38 (58.5) | .51 |
| *1h - 2h* | 17 (16.2) | 11 (16.9) | 1.0 |
| *30min - 1h* | 14 (13.3) | 12 (18.5) | .49 |
| *<30min* | 6 (5.7) | 4 (6.1) | 1.0 |
| **Awakenings due to pain***, n (%)* |  |  |  |
| *Several times a night* | 25 (23.8) | 15 (23.1) | 1.0 |
| *Once a night* | 20 (19.0) | 11 (16.9) | .88 |
| *Sometimes* | 53 (50.5) | 33 (50.8) | 1.0 |
| *Never* | 7 (6.7) | 6 (9.2) | .56 |
| **Type of analgesic drugs***, n (%)* |  |  |  |
| *Local analgesics / anesthetics or patch* | 14/74 (18.9) | 5/38 (13.2) | .61 |
| *Antidepressants* | 54/74 (73.0) | 24/38 (63.2) | .39 |
| *Antiepileptics* | 28/74 (37.8) | 17/38 (44.7) | .62 |
| *Weak opioids ± paracetamol* | 49/74 (66.2) | 25/38 (65.8) | 1.0 |
| *Strong opioids* | 14/74 (18.9) | 5/38 (13.2) | .61 |
| *Nonsteroidal anti-inflammatory drug* | 17/74 (23.0) | 5/38 (13.2) | .32 |
| *Paracetamol (without opioid associations)* | 18/74 (24.3) | 11/38 (28.9) | .76 |
| *Benzodiazepines* | 16/74 (21.6) | 5/38 (13.2) | .41 |
| *Others (nefopam, cannabis, levocarnitine…)* | 23/74 (31.1) | 11/38 (28.9) | .99 |
| **Use of non-medicinal analgesic techniques***, n (%)* | 66/74 (89.2) | 33/38 (86.8) | .76 |

**SUPPLEMENTARY TABLE 1 – CHARACTERIZATION OF THE STUDY POPULATIONS.** Characteristics of included patients and patients who completed the study (at least 3 months of follow-up).

|  | **CLUSTERS** | | | |  |  |
| --- | --- | --- | --- | --- | --- | --- |
|  | **1** *n=37* | **2** *n=14* | **3** *n=23* | **4** *n=30* | ***Effect-size*** | ***p value*** |
| **BASELINE SOCIO-DEMOGRAPHIC CHARACTERIZATION** | | | | | | |
| **Female gender**, n (%) | 26 (70.3) | 11 (78.6) | 19 (82.6) | 24 (80.0) | 0.12 [0.00;0.25] | .68 |
| **Age** (years), med [95% CI] | 47.0 [37.0;56.0] | 50.0 [42.8;59.0] | 47.0 [42.0;56.0] | 46 [38.0;51.0] | -0.02 [-0.03;0.07] | .71 |
| *18 to 39 years, n (%)* | 13 (35.1) | 3 (21.4) | 5 (21.7) | 9 (30.0) | 0.13 [0.00;0.31] | .73 |
| *40 to 59 years, n (%)* | 18 (48.7) | 7 (50.0) | 14 (60.9) | 19 (63.3) |
| *≥ 60 years, n (%)* | 6 (16.2) | 4 (28.6) | 4 (17.4) | 2 (6.7) |
| ***Children*** *(NO), n (%)* | 15 (40.5) | 7 (50.0) | 11 (47.8) | 15 (50.0) | 0.09 [0.00;0.22] | .86 |
| **Single**, n (%) | 10 (27.0) | 3 (21.4) | 7 (30.4) | 9 (30.0) | 0.07 [0.00;0.21] | .93 |
| **Level of education**, n (%) |  |  |  |  |  |  |
| *A level or less* | 16 (43.2) | 8 (57.1) | 9 (39.1) | 19 (63.3) | 0.16 [0.04;0.27] | .53 |
| *Diploma of Higher Education and BA, BS/BSc* | 12 (32.4) | 4 (28.6) | 10 (43.5) | 7 (23.3) |
| *MS/MSc, MA or more* | 9 (24.3) | 2 (14.3) | 4 (17.4) | 4 (13.3) |
| **Profession**, n (%) |  |  |  |  |  |  |
| *Craftsmen, merchants, entrepreneurs, executives and senior professionals* | 9 (24.3) | 0 (0.0) | 5 (21.7) | 4 (13.3) | 0.27 [0.18;0.35] | .10 |
| *Employees, factory workers and intermediate professions* | 18 (48.7) | 4 (28.6) | 14 (60.9) | 14 (46.7) |
| *Without professional activity* | 10 (27.0) | 10 (71.4) | 4 (17.4) | 12 (40.0) |
| **Professional situation**, n (%) |  |  |  |  |  |  |
| *In activity* | 31 (83.8) | 12 (85.7) | 21 (91.3) | 20 (66.7) | 0.19 [0.08;0.29] | .13 |
| *Work stoppage due to pain* | 7/31 (22.6) | 3/12 (25.0) | 12/21 (57.1) | 8/20 (40.0) |
| *Disability or Disabled adult allowance* | 7/31 (22.6) | 8/12 (66.7) | 4/21 (19.0) | 7/20 (35.0) |
| *Unemployed* | 4 (10.8) | 1 (7.1) | 1 (4.3) | 9 (30.0) |
| *Student* | 1 (2.7) | 0 (0.0) | 0 (0.0) | 0 (0.0) |
| *Retired* | 1 (2.7) | 1 (7.1) | 2 (8.7) | 4 (13.3) |
| **Tobacco***, Smoker, n (%)* | 12 (32.4) | 0 (0.0) | 5 (21.7) | 10 (33.3) | 0.26 [0.16;0.35] | .08 |
| **Alcohol***, Drinker, n (%)* | 22 (59.4) | 6 (42.9) | 16 (693.6) | 18 (60.0) | 0.16 [0.01;0.31] | .46 |
| **BASELINE QUESTIONNARIES SCORES** | | | | | | |
| **Total score EPICES**, med [interquartile range] | 18.0 [7.0;31.0] | 16.5 [7.3;24.8] | 40.0 [15.0;56.0] | 37.0 [21.0;49.0] | 0.13 [0.03;0.25] | **.02^b,c,d,e^** |
| *% precarious (score ≥30), n (%)* | 9/33 (27.3) | 2 (14.3) | 11/17 (64.7) | 18/29 (62.1) | 0.43 [0.25;0.60] | **<.001^b,c,d,e^** |
| **Total score TSK** (/68), med [interquartile range] | 36.0 [33.3;41.8] | 44.0 [41.0;48.0] | 46.0 [37.0;50.0] | 49.0 [45.3;51.0] | 0.34 [0.19;0.48] | **<.001^a,c^** |
| *% score ≥37 (proven kinesiophobia), n (%)* | 15/34 (44.1) | 12/13 (92.3) | 13/17 (76.5) | 27/28 (96.4) | 0.52 [0.36;0.68] | **<.001^a,b,c^** |
| **Total score IEQ** (/48), med [interquartile range] | 18.5 [12.0;24.3] | 34.0 [23.0;36.5] | 31.0 [27.8;36.8] | 37.0 [32.5;40.8] | 0.45 [0.32;0.58] | **<.001^a,b,c^** |
| **Total score TAS20** (/100), med [interquartile range] | 52.5 [47.0;62.0] | 64.5 [58.5;70.8] | 57.5 [52.8;65.0] | 68.0 [61.3;73.0] | 0.25 [0.08;0.39] | **<.001^c^** |
| *% score ≥61 (proven alexithymia), n (%)* | 10/36 (27.8) | 9 (64.3) | 9/20 (45.0) | 23 (76.7) | 0.41 [0.23;0.59] | **<.001^a,c,d,f^** |
| **BFI-44** (score /5), med [interquartile range] |  |  |  |  |  |  |
| *Extraversion* | 2.8 [2.5;3.1] | 2.5 [2.2;3.0] | 3.1 [2.6;3.3] | 2.2 [1.9;2.7] | 0.27 [0.13;0.43] | **<.001^a,c,f^** |
| *Agreeableness* | 4.4 [4.1;4.6] | 3.8 [3.5;4.1] | 4.2 [4.0;4.5] | 4.0 [3.5;4.5] | 0.14 [0.04;0.31] | **<.001^c,f^** |
| *Conscientiousness* | 3.9 [3.6;4.1] | 3.2 [2.9;3.7] | 4.0 [3.7;4.2] | 3.4 [3.1;3.7] | 0.16 [0.06;0.32] | **<.001^c,f^** |
| *Neuroticism* | 2.2 [1.7;2.7] | 2.9 [2.7;3.3] | 2.5 [1.9;2.9] | 3.2 [2.9;3.6] | 0.17 [0.03;0.36] | **<.001^a,c,d,f^** |
| *Openness to experience* | 3.7 [3.4;4.1] | 3.6 [3.1;4.1] | 3.9 [3.8;4.4] | 3.0 [2.8;3.7] | 0.12 [0.05;0.29] | **.002^a,c,d^** |
| **MOS-SS** |  |  |  |  |  |  |
| *% optimal sleep (7-8h / night), n (%)* | 16/34 (47.1) | 6/13 (46.1) | 3/18 (16.7) | 5/28 (17.9) | 0.31 [0.13;0.50] | **.03** |
| *Sleep Problem Index (SPI),* med [interquartile range] | 42.5 [36.0;59.3] | 49.0 [43.0;56.0] | 65.0 [56.0;72.5] | 62.5 [53.5;73.0] | 0.11 [0.03;0.27] | **.002^b,c^** |
| **HADS** |  |  |  |  |  |  |
| *Total score anxiety,* med [interquartile range] | 6.0 [4.0;8.0] | 10.0 [7.0;12.0] | 8.5 [8.0;12.0] | 13.0 [10.0;16.0] | 0.33 [0.21;0.50] | **<.001^b,c,e^** |
| No symptom (score <7), n (%) | 19/34 (55.9) | 2/13 (15.4) | 2/18 (11.1) | 0/28 (0.0) |  | **<.001^a,b,c,e,f^** |
| Borderline (score ≥7 to <11), n (%) | 10/34 (29.4) | 7/13 (53.8) | 9/18 (50.0) | 9/28 (32.1) | 0.44 [0.32;0.56] |
| Clinical caseness (score ≥11), n (%) | 5/34 (14.7) | 4/13 (30.8) | 7/18 (38.9) | 19/28 (67.9) |  |
| *Total score depression,* med [interquartile range] | 4.5 [3.0;8.0] | 6.0 [4.0;9.0] | 8.5 [6.5;11.0] | 11.0 [8.8;14.0] | 0.31 [0.22;0.45] | **<.001^b,c,e^** |
| No symptom (score <7), n (%) | 23/34 (67.6) | 7/13 (53.8) | 5/18 (27.8) | 1/28 (3.6) |  | **<.001^b,c,e,f^** |
| Borderline (score ≥7 to <11), n (%) | 9/34 (26.5) | 4/13 (30.8) | 7/18 (38.9) | 12/28 (42.9) | 0.42 [0.33;0.52] |
| Clinical caseness (score ≥11), n (%) | 2/34 (5.9) | 2/13 (15.4) | 6/18 (33.3) | 15/28 (53.6) |  |
| **Total score PCS** (/52), med [interquartile range] | 19.5 [10.5;28.0] | 28.5 [24.5;32.0] | 31.0 [24.0;37.0] | 37.0 [33.0;41.8] | 0.40 [0.26;0.54] | **<.001^b,c,e^** |
| *% score ≥30 (proven catastrophism)*, n (%) | 6/36 (16.7) | 5 (35.7) | 11/19 (57.9) | 25 (83.3) | 0.56 [0.42;0.70] | **<.001^a,b,c,e,f^** |
| **Total score SWLS** (/35), med [interquartile range] | 22.0 [18.0;27.0] | 18.0 [16.0;20.0] | 22.0 [17.0;25.0] | 14.0 [11.0;18.0] |  | **<.001^c^** |
| *% score ≤19 (degraded life satisfaction)*, n (%) | 11/33 (33.3) | 9 (64.3) | 7/16 (43.7) | 24/29 (82.6) | 0.42 [0.26;0.59] | **<.001^a,c,f^** |
| **Health status - EQ-5D-3L** (/100), med [interquartile range] | 60.0 [45.0;70.0] | 60.0 [53.0;65.0] | 41.5 [29.0;51.8] | 48.5 [28.3;63.5] | 0.07 [-0.01;0.20] | **.02^b,d^** |
| **Total score LOT-R** (/24), med [interquartile range] | 13.0 [12.0;14.0] | 11.5 [11.0;14.0] | 11.0 [10.0;12.0] | 11.0 [10.0;12.0] | 0.13 [0.03;0.31] | **<.001^b,c^** |
| **Total score BJW** (/30), med [interquartile range] | 19.0 [17.0;22.0] | 18.5 [14.0;21.0] | 15.0 [14.0;18.0] | 16.0 [12.0;17.8] | 0.18 [0.07;0.35] | **<.001^b,c,e^** |
| **PBPI** (score -2 to +2), med [interquartile range] |  |  |  |  |  |  |
| *Mystery* | -1.0 [-1.40;-0.40] | 0.20 [-0.60;0.20] | -0.20 [-0.50;0.00] | 0.20 [-0.40;0.40] | 0.26 [0.13;0.43] | **<.001^a,b,c^** |
| *Chronicity* | 1.0 [0.67;1.67] | -0.33 [-0.58;0.50] | 0.83 [0.00;1.33] | 0.00 [-0.33;0.33] | 0.22 [0.08;0.40] | **<.001^a,c,d,f^** |
| *Constancy* | 0.0 [-0.25;0.25] | 0.25 [0.25;0.94] | 0.38 [0.00;0.75] | -0.25 [-0.75;0.00] | 0.28 [0.16;0.44] | **<.001^a,b,c,e,f^** |
| *Self-Blame* | 0.33 [0.33;1.00] | 0.67 [0.42;0.92] | 0.67 [0.67;1.00] | 0.67 [-0.33;0.67] | 0.06 [0.01;0.20] | **.04** |
| **SCC**, *possible cognitive disorders*, n (%) | 21/32 (65.6) | 11 (78.6) | 16/16 (100) | 29 (96.7) | 0.26 [0.12;0.46] | **<.001^b,c^** |
| **BASELINE PAIN DISORDERS CHARACTERIZATION** | | | | | | |
| **BPI** |  |  |  |  |  |  |
| *General pain intensity (/10),* med [interquartile range] | 6.0 [5.0;7.0] | 7.0 [6.0;7.8] | 7.0 [6.0;7.5] | 6.5 [5.0;8.0] | 0.05 [-0.01;0.19] | .20 |
| *<4/10 (mild pain), n (%)* | 3/27 (11.1) | 2/12 (16.7) | 0/15 (0.0) | 2/28 (7.1) | 0.18 [0.06;0.30] | .54 |
| *≥4/10 and <7/10 (moderate pain), n (%)* | 14/27 (51.9) | 3/12 (25.0) | 6/15 (40.0) | 12/28 (42.9) |
| *≥7/10 (severe pain), n (%)* | 10/27 (37.0) | 7/12 (58.3) | 9/15 (60.0) | 14/28 (50.0) |
| *Total score pain interference (/10),* med [interquartile range] | 3.3 [2.2;4.1] | 4.1 [3.6;4.6] | 4.8 [4.1;5.4] | 4.4 [3.6;5.0] | 0.11 [0.03;0.30] | **.002^b,c^** |
| *High impact chronic pain, n (%)* | 3/27 (11.1) | 0/12 (0.0) | 7/15 (46.7) | 7/28 (25.0) | 0.37 [0.17;0.56] | **.01^b,c^** |
| **Type of pain***, n (%)* |  |  |  |  |  |  |
| *Neuropathic (MG30.50/51 and others)* | 6/29 (20.7) | 3/11 (27.3) | 5/20 (25.0) | 4/19 (21.0) | .19 | .45 |
| *Nociceptive (MG30.20/21)* | 4/29 (13.8) | 1/11 (9.1) | 3/20 (15.0) | 0/19 (0.0) |
| *Nociplastic (MG30.00/01/02/42 and 8A80.1/2)* | 15/29 (51.7) | 4/11 (36.4) | 11/20 (55.0) | 14/19 (73.7) |
| *Others* | 4/29 (13.8) | 3/11 (27.3) | 1/20 (5.0) | 1/19 (5.3) |
| **Pain seniority***, n (%)* |  |  |  |  |  |  |
| *≤3 years* | 7 (18.9) | 2 (14.3) | 4 (17.4) | 12 (40.0) | 0.18 [0.06;0.30] | .39 |
| *4-5 years* | 8 (21.6) | 4 (28.6) | 6 (26.1) | 7 (23.3) |
| *>5 years* | 22 (59.5) | 8 (57.1) | 13 (56.5) | 11 (36.7) |
| *^a^ cluster 1 vs 2; ^b^ cluster 1 vs 3; ^c^ cluster 1 vs 4; ^d^ cluster 2 vs 3; ^e^ cluster 2 vs 4; ^f^ cluster 3 vs 4* | | | | | | |

**SUPPLEMENTARY TABLE 2 – CLUSTERING OF INCLUDED PATIENTS.** Characteristics of the 4 patient subgroups (cluster). Percentages should be read in columns. For example, in cluster 1, 35.1% of the patients are between 18 and 39 years old.

|  | **Adherent patient** *n=105* | |  |  |
| --- | --- | --- | --- | --- |
|  | **FALSE** | **TRUE** | ***Effect size*** | ***p*-value** |
| **BASELINE SOCIO-DEMOGRAPHIC CHARACTERIZATION** | | | | |
| **Gender**, n (%) |  |  |  |  |
| *Female* | 31/81 (38.3) | 50/81 (61.7) | 0.07 [0.00;0.26] | .47 |
| *Male* | 12/24 (50.0) | 12/24 (50.0) |
| **Marital status**, n (%) |  |  |  |  |
| *Single* | 10/29 (34.5) | 19/29 (65.5) | 0.06 [0.00;0.25] | .35 |
| *Couple* | 33/76 (43.4) | 43/76 (56.6) |
| **Children**, n (%) |  |  |  |  |
| *Yes* | 23/49 (46.9) | 26/49 (53.1) | 0.09 [0.00;0.29] | .32 |
| *No* | 20/56 (35.7) | 36/56 (64.3) |
| **Level of education**, n (%) |  |  |  |  |
| *A level or less* | 21/52 (40.4) | 31/52 (59.6) | 0.04 [0.00;0.15] | .92 |
| *Diploma of Higher Education and BA. BS/BSc* | 13/33 (39.4) | 20/33 (60.6) |
| *MS/MSc. MA or more* | 9/20 (45.0) | 11/20 (55.0) |
| **Profession**, n (%) |  |  |  |  |
| *Craftsmen, merchants, entrepreneurs, executives and Senior Professionals* | 7/19 (36.8) | 12/19 (63.2) | 0.24 [0.00;0.36] | .39 |
| *Employees, factory workers and Intermediate professions* | 18/50 (36.0) | 32/50 (64.0) |
| *Without professional activity* | 18/36 (50.0) | 18/36 (50.0) |
| **Professional situation**, n (%) |  |  |  |  |
| *In activity* | 32/85 (37.6) | 53/85 (62.3) | 0.17 | .38 |
| *Work stoppage due to pain* | 10/30 (33.3) | 20/30 (66.7) |
| *Disability or Disabled adult allowance* | 9/26 (34.6) | 17/26 (65.4) |
| *Unemployed* | 8/15 (53.3) | 7/15 (46.7) |
| *Student* | 1/1 (100.0) | 0/1 (0.0) |
| *Retired* | 2/4 (50.0) | 2/4 (50.0) |
| **Age**, n (%) |  |  |  |  |
| *18 to 39 years* | 9/18 (50.0) | 9/18 (50.0) | 0.09 | .61 |
| *40 to 59 years* | 22/58 (37.9) | 36/58 (62.1) |
| *≥ 60 years* | 13/30 (43.3) | 17/30 (56.7) |
| **Tobacco**, n (%) |  |  |  |  |
| YES | 10/27 (37.0) | 17/27 (63.0) | 0.02 [0.00;0.20] | .66 |
| NO | 33/78 (42.3) | 45/78 (57.7) |
| **Alcohol**, n (%) |  |  |  |  |
| *YES* | 28/62 (45.2) | 34/62 (54.8) | 0.08 [0.00;0.27] | .39 |
| *NO* | 15/43 (34.9) | 28/43 (65.1) |
| **BASELINE PAIN DISORDERS CHARACTERIZATION** | | | | |
| **Type of pain**, n (%) |  |  |  |  |
| *Neuropathic (MG30.50/51 and others)* | 10/18 (55.6) | 8/18 (44.4) | 0.37 | **.01^a^** |
| *Nociceptive (MG30.20/21)* | 5/8 (62.5) | 3/8 (37.5) |
| *Nociplastic (MG30.00/01/02/42 and 8A80.1/2)* | 15/45 (33.3) | 30/45 (66.7) |
| *Others* | 8/9 (88.9) | 1/9 (11.1) |
| **Pain seniority**, n (%) |  |  |  |  |
| *> 5 years* | 24/55 (43.6) | 31/55 (56.4) | 0.15 [0.00;0.32] | .30 |
| *4 – 5 years* | 12/25 (48.0) | 13/25 (62.0) |
| ≥ *6 months and < 4 years* | 7/25 (28.0) | 18/25 (72.0) |
| **Pain frequency**, n (%) |  |  |  |  |
| *Permanent (all day)* | 20/56 (35.7) | 36/56 (64.3) | 0.12 [0.00;0.25] | .66 |
| *Very frequent (every day)* | 19/40 (47.5) | 21/40 (52.5) |
| *Frequent (several times a week)* | 3/7 (42.9) | 4/7 (57.1) |
| *Rare (less than once a week)* | 1/2 (50.0) | 1/2 (50.0) |
| **Painful attacks frequency**, n (%) |  |  |  |  |
| *Very frequent (several times a day)* | 13/30 (43.3) | 17/30 (56.7) | 0.09 [0.00;0.21] | .96 |
| *Frequent (daily)* | 16/41 (39.0) | 25/41 (61.0) |
| *Rare (not every day)* | 14/33 (42.4) | 19/33 (57.6) |
| *Exceptional (never or almost never)* | 0/1 (0.0) | 1/1 (100.0) |
| **Pain attacks duration**, n (%) |  |  |  |  |
| ≥ *2h* | 31/68 (45.6) | 37/68 (54.4) | 0.17 [0.00;0.32] | .39 |
| ≥ *1h and < 2h* | 7/17 (41.2) | 10/17 (58.8) |
| ≥ *30 min and < 1h* | 3/14 (21.4) | 11/14 (78.6) |
| *< 30 min* | 2/6 (33.3) | 4/6 (66.7) |
| **Number of awakenings per night due to pain**, n (%) |  |  |  |  |
| *Several times* | 11/25 (44.0) | 14/25 (56.0) | 0.11 [0.00;0.24] | .61 |
| *At least once* | 10/20 (50.0) | 10/20 (50.0) |
| *Sometimes* | 19/53 (35.8) | 34/53 (64.2) |
| *Never* | 3/7 (42.9) | 4/7 (57.1) |
| **BASELINE QUESTIONNAIRES SCORES** | | | | |
| **QCD** |  |  |  |  |
| *Total score*, med [interquartile range] | 62.0 [39.6;83.2] | 63.0 [34.6;85.4] | -0.01 [-0.01;0.07] | .58 |
| *General pain intensity*, med [interquartile range] | 7.0 [4.2;10] | 6.0 [3.6;8.3] | -0.01 [-0.01;0.04] | .63 |
| *Interference*, med [interquartile range] | 36.0 [23.6;47.2] | 39.5 [18.6;56.7] | -0.01 [-0.01;0.07] | .63 |
| *High impact chronic pain, n (%)* |  |  |  |  |
| *YES* | 5/17 (29.4) | 12/17 (70.6) | 0.01 [0.00;0.18] | .92 |
| *NO* | 26/76 (34.2) | 50/76 (65.8) |
| **HADS-Anxiety** |  |  |  |  |
| *No symptom (score <7)*, n (%) | 10/24 (41.7) | 14/24 (58.3) | 0.11 [0.00;0.28] | .58 |
| *Borderline (score ≥7 to <11)*, n (%) | 10/35 (28.6) | 25/35 (71.4) |
| *Clinical caseness (score ≥11)*, n (%) | 12/35 (34.3) | 23/35 (65.7) |
| **HADS-Depression** |  |  |  |  |
| *No symptom (score <7)*, n (%) | 13/36 (36.1) | 23/36 (63.9) | 0.05 [0.00;0.17] | .91 |
| *Borderline (score ≥7 to <11)*, n (%) | 11/32 (34.4) | 21/32 (65.6) |
| *Clinical caseness (score ≥11)*, n (%) | 8/26 (30.8) | 18/26 (69.2) |
| **PBPI** (score -2 to +2), med [interquartile range] |  |  |  |  |
| *Self-blame* | 0.33 [-0.67;1.45] | 0.67 [-1.00;1.33] | -0.01 [-0.01;0.05] | .98 |
| *Mystery* | -0.60 [-1.20;0.48] | -0.20 [-1.80;0.67] | -0.01 [-0.01;0.05] | .98 |
| *Chronicity* | 0.00 [-0.60;2.00] | 0.50 [-0.79;2.00] | 0.01 [-0.01;0.11] | .13 |
| *Constancy* | -0.25 [-1.05;0.95] | 0.00 [-1.00;1.00] | 0.00 [-0.01;0.08] | .30 |
| **MOS-Sleep Scale**, *SPI,* med [interquartile range] | 52.0 [33.0;75.0] | 59.5 [27.9;80.0] | 0.01 [-0.01;0.09] | .15 |
| **SCC**, *possible cognitive disorders*, n (%) |  |  |  |  |
| YES | 22/77 (28.6) | 55/77 (71.4) | 0.03 [-0.01;0.13] | **.04** |
| NO | 9/16 (56.3) | 7/16 (43.7) |
| **Total Score BJW** (/30)*,* med [interquartile range] | 18.0 [9.8;23.4] | 17.0 [11.0;23.4] | -0.01 [-0.01;0.04] | .82 |
| **Total score LOT-R** (/24), med [interquartile range] | 12.0 [10.6;14.0 | 12.0 [8.7;14.4] | 0.00 [-0.01;0.06] | .47 |
| **Total score IEQ** (/48), med [interquartile range] | 29.0 [12.0;42.2] | 31.5 [12.3;44.0] | 0.01 [-0.01;0.10] | .13 |
| **Health status-EQ-5D-3L**(/100), med [interquartile range] | 64.0 [8.0;84.6] | 50.5 [15.0;78.7] | -0.01 [-0.01;0.06] | .60 |
| **PCS**, *score ≥30*, n (%) |  |  |  |  |
| YES | 14/47 (29.8) | 33/47 (70.2) | 0.14 [0.00;0.33] | .16 |
| NO | 24/53 (45.3) | 29/53 (54.7) |
| **TSK**, s*core ≥37*, n (%) |  |  |  |  |
| YES | 19/67 (28.4) | 48/67 (71.6) | 0.12 [0.00;0.33] | .24 |
| NO | 11/25 (44.0) | 14/25 (56.0) |
| **SWLS**, *score < 20*, n (%) |  |  |  |  |
| YES | 15/51 (29.4) | 36/51 (70.6) | 0.08 [0.00;0.28] | .45 |
| NO | 16/41 (39.0) | 25/41 (61.0) |
| **EPICES**, *score ≥30*, n (%) |  |  |  |  |
| YES | 10/39 (25.6) | 29/39 (74.4) | 0.13 [0.00;0.34] | .20 |
| NO | 22/54 (40.7) | 32/54 (59.3) |
| **TAS-20**, score > 60, n (%) |  |  |  |  |
| YES | 14/51 (27.4) | 37/51 (72.6) | 0.20 [0.00;0.40] | **.04** |
| NO | 24/49 (49.0) | 25/49 (51.0) |
| **CLUSTERING** | | | | |
| **1**, n (%) | 18 (41.9) | 19 (30.6) | 0.29 [0.00;0.46] | **.03^b,c^** |
| **2**, n (%) | 5 (11.6) | 9 (14.5) |
| **3**, n (%) | 13 (30.2) | 10 (16.1) |
| **4**, n (%) | 6 (14.0) | 24 (38.7) |
| *^a^ nociplastic vs others; ^b^ cluster 1 vs 4; ^c^ cluster 3 vs 4* | | | | |

**SUPPLEMENTARY TABLE 3 - CHARACTERIZATION OF ADHERENT PATIENTS.** Characteristics of adherent and non-adherent patients to the eDOL tool. The percentages should be read in a row. For example, 61.7% of women are eDOL adherent.
